# A conserved capsid-based multi-epitope vaccine targeting dengue virus serotypes (DENV1–4): an integrated computational and in vivo study

**DOI:** 10.1186/s12865-026-00857-1

**Published:** 2026-05-27

**Authors:** Elham Mohammed Khatrawi, Syed Luqman Ali, Awais Ali, Syed Mudasser Ali, Hind Althagafi

**Affiliations:** 1 Department of Basic Medical Sciences, College of Medicine, Taibah University, Madinah, 42353 Saudi Arabia; 2 Department of Biochemistry Abdul, Wali Khan University Mardan, Mardan, KPK 23200 Pakistan; 3 Department of Medical Laboratory Sciences, Abasyn University, Peshawar, KPK Pakistan; 4 Department of Biology, College of Science, Princess Nourah bint Abdulrahman University, P.O. Box 84428, Riyadh, 11671 Saudi Arabia

**Keywords:** Dengue Virus Serotypes (DENV), Multi-epitope vaccine (MEV), Reverse vaccinology, Capsid protein, Immunoinformatics, B-cell epitopes, T-cell epitopes, Molecular docking, Immune simulation, *In vivo*

## Abstract

**Background:**

Dengue virus (DENV), comprising four antigenically distinct serotypes (DENV1–4), continues to pose a major global health burden. The lack of a universally protective vaccine capable of inducing balanced immunity against all serotypes remains a critical challenge, largely due to antigenic diversity and the risk of antibody-dependent enhancement (ADE). Conserved capsid proteins represent promising targets for epitope-based vaccine development because of their structural stability and immunogenic potential.

**Methods:**

An integrated reverse vaccinology and immunoinformatics strategy was employed to design a conserved capsid-based multi-epitope vaccine (MEV-DV) targeting DENV1–4. B-cell, cytotoxic T-lymphocyte (CTL), and helper T-lymphocyte (HTL) epitopes were screened for antigenicity, non-allergenicity, non-toxicity, and sequence conservancy. Selected epitopes were assembled using appropriate linkers with β-defensin as an adjuvant and a PADRE sequence. The construct was evaluated for physicochemical properties, 3D structure modeling and validation, solubility, disulfide engineering, molecular docking with TLR4 and TLR8, molecular dynamics simulation, and immune simulation. Experimental validation was performed in albino mice using an alum-adjuvanted formulation, and humoral responses were assessed by hemagglutination inhibition (HI) assay.

**Results:**

The finalized MEV-DV construct exhibited high antigenicity (0.8559), favorable physicochemical properties, and structural stability, with 97.6% of residues located in favored Ramachandran regions. Docking studies demonstrated strong binding affinity with TLR4 and TLR8, and molecular dynamics simulations confirmed stable receptor–vaccine interactions over 50 ns. Immune simulations predicted robust humoral and cellular immune responses with sustained memory formation. In vivo immunization induced detectable antibodies by day 7, with peak HI titers at day 21. Antibody levels were statistically comparable to those elicited by a commercial inactivated dengue vaccine (*p* > 0.05), and no adverse effects were observed.

**Conclusion:**

This study demonstrates that a conserved capsid-based multi-epitope vaccine designed through reverse vaccinology and immunoinformatics is structurally stable, immunogenic, and safe in a murine model. The combined computational and experimental findings support the potential of MEV-DV as a promising broadly protective dengue vaccine candidate and warrant further evaluation through neutralization assays and viral challenge studies.

**Graphical Abstract:**

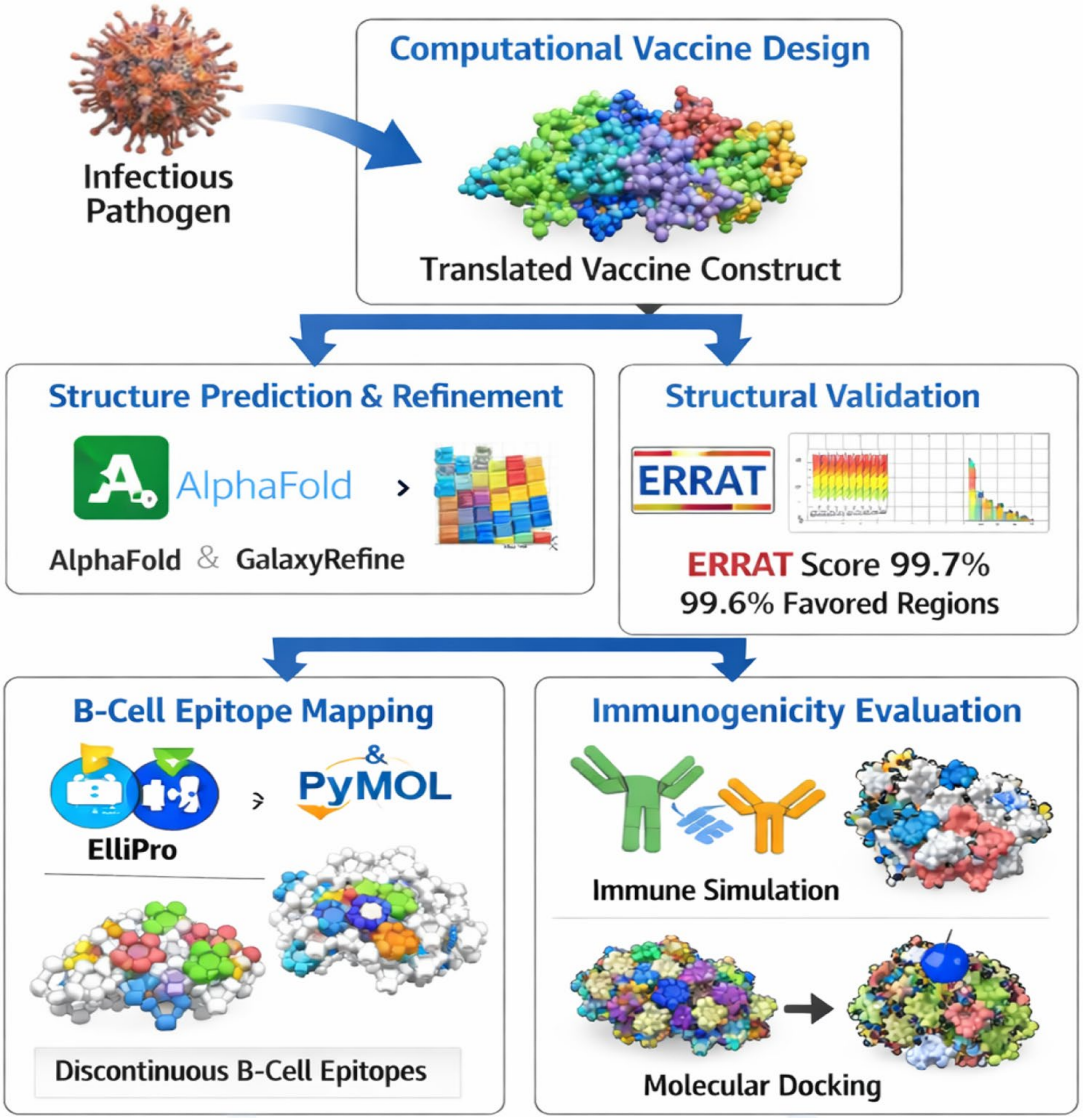

**Supplementary Information:**

The online version contains supplementary material available at 10.1186/s12865-026-00857-1.

## Introduction

Dengue is a vector-borne viral disease caused by the DENV, which belongs to the Flavivirus genus of the Flaviviridae family [1]. The virus is transmitted to humans predominantly through the bites of infected female Aedes aegypti mosquitoes, and to a lesser extent, Aedes albopictus. Dengue is a major public health concern in tropical and subtropical regions, with an estimated 390 million infections occurring annually worldwide [2]. The disease presents a spectrum of clinical symptoms, ranging from mild dengue fever characterized by fever, rash, and joint pain, to severe forms such as dengue hemorrhagic fever (DHF) and dengue shock syndrome (DSS), which can lead to organ failure, internal bleeding, and death if untreated [3]. A unique challenge in combating dengue lies in its four genetically and antigenically distinct serotypes: DENV1, DENV2, DENV3, and DENV4 [4]. Each serotype has the ability to cause disease independently, and recovery from one serotype provides lifelong protection against that specific type but offers no lasting protection against the others [5].

Dengue fever, caused by any of the four serotypes of Dengue virus (DENV-1 to DENV-4), represents one of the most pervasive and rapidly expanding vector-borne zoonotic diseases globally, with an estimated hundreds of millions of infections annually and a wide spectrum of clinical outcomes from mild febrile illness to severe hemorrhagic manifestations. Dengue’s emergence and persistence are closely linked to complex interactions among humans, vectors (primarily *Aedes* mosquitoes), animal hosts, and environmental factors, reflecting the intricate dynamics of zoonotic spillover and adaptation from sylvatic cycles into urban transmission. Recent genomic studies have provided evidence of sporadic spillovers of sylvatic DENV lineages into humans, highlighting the evolutionary continuum between wildlife reservoirs and epidemic strains and underscoring the gap in current surveillance tools to detect atypical variants. These characteristics place dengue firmly within the broader category of emerging zoonotic diseases that are influenced by anthropogenic changes, global travel, climate variability, and urbanization, all of which can expand vector habitats and facilitate epidemic spread. Addressing the multifaceted challenge of dengue thus requires a coordinated One Health approach that integrates human and animal health surveillance, vector ecology, and environmental monitoring to enable early detection, effective response, and sustainable control strategies. Within this context, novel vaccine design efforts—particularly those targeting conserved antigens across all serotypes are critically needed to complement integrated surveillance and control measures, mitigate disease burden, and contribute to long-term public health resilience.

**Fig. 1:**
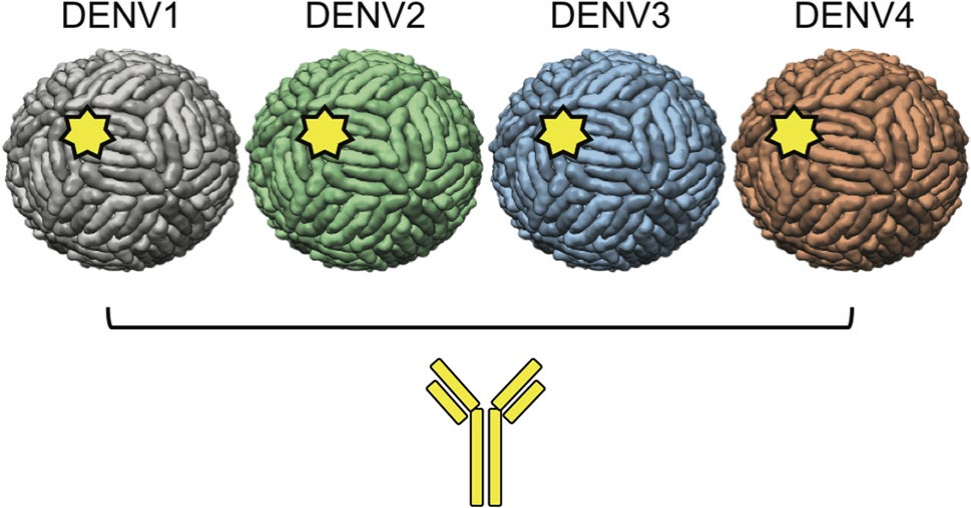
A cross-reactive antibody targeting a conserved epitope (yellow star) shared across all four dengue virus serotypes

**Fig. 2:**
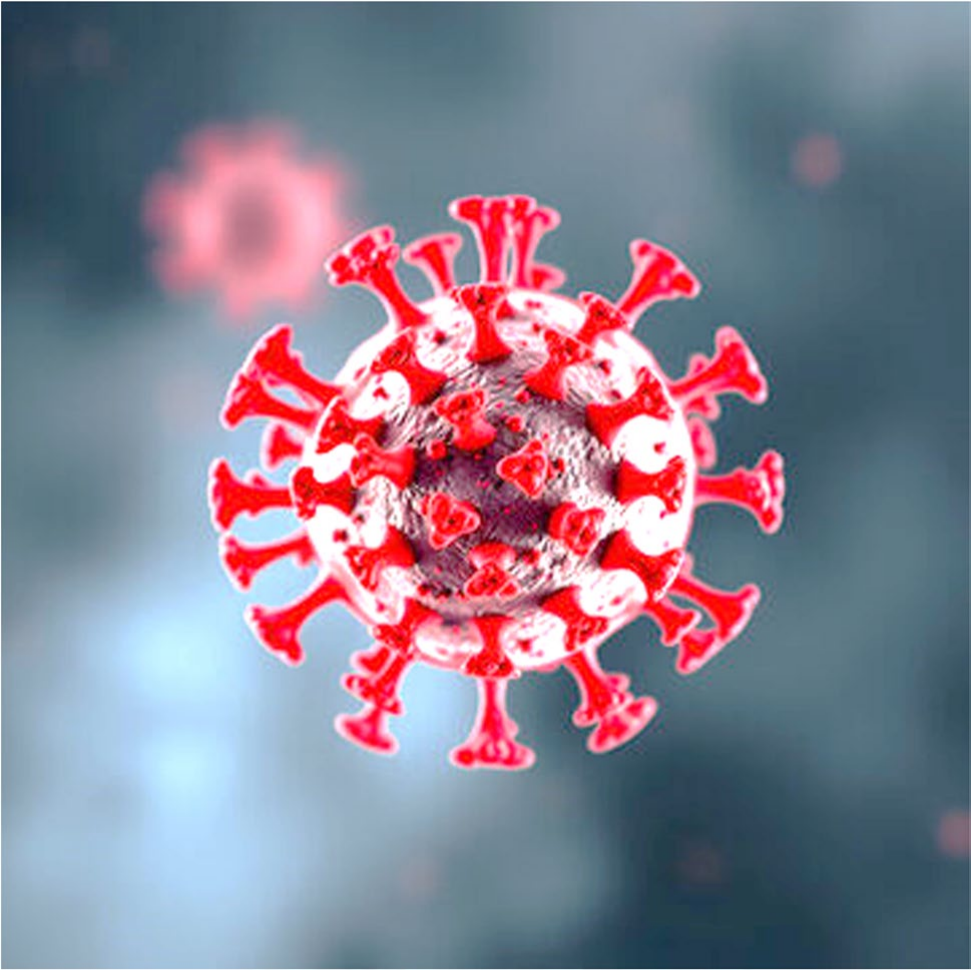
Morphology of dengue virus serotype 4

These four serotypes contribute to the complexity of dengue epidemiology. While a primary infection with one serotype is often mild or moderate, subsequent infections with a different serotype can result in severe disease due to a phenomenon called ADE [6]. In this process, pre-existing, non-neutralizing antibodies from a prior infection facilitate increased viral entry into host cells, leading to higher viral loads and more severe immune responses. Among the serotypes, DENV2 and DENV3 are historically associated with more severe disease outbreaks, but all four can lead to severe cases under certain conditions [7]. DENV4, in particular, while often considered less common in some regions, has been increasingly implicated in significant outbreaks (Fig. 1). This serotype presents unique challenges due to its genetic variability and the potential to cause severe disease in secondary infections (Fig. 2) [8].

The absence of a fully effective, universally protective vaccine against Dengue virus serotypes 1, 2, 3, and 4 continues to hinder global dengue control efforts. Infections caused by Dengue virus type 1, Dengue virus type 2, Dengue virus type 3, and Dengue virus type 4 can result in a broad clinical spectrum, ranging from self-limiting febrile illness to severe dengue characterized by plasma leakage, hemorrhage, and organ dysfunction. Currently, there is no approved antiviral therapy that directly targets these serotypes, and clinical management relies primarily on supportive care, which may be insufficient in severe cases, particularly in resource-constrained settings. Moreover, the development of a merged or universal vaccine has been exceptionally challenging due to the need to induce balanced and long-lasting immunity against all four serotypes simultaneously, as incomplete or unbalanced immune responses may increase the risk of antibody-dependent enhancement (ADE) during secondary infections. In this research, we aim to address this critical gap by designing targeted immunogenic strategies that comprehensively consider the antigenic diversity and evolutionary dynamics of all four serotypes. By integrating advanced molecular biology, immunoinformatics, and genomic analyses, we focus on identifying conserved and serotype-specific epitopes capable of eliciting robust and protective immune responses. Our ultimate goal is to develop a safe, broadly protective, and scalable vaccine candidate that can effectively reduce the incidence, severity, and global burden of dengue caused by DENV1–4 [9].

## Methodology

### Retrieval of capsid protein sequence of dengue type 1, 2, 3 and 4

The three Capsid protein sequence of DENV1, DENV2, DENV3 and DENV4 are downloaded from NCBI database following accession numbers NP_059433.1, NP_739591.2, A0A217EIR2, ARO34439.1, ARO34441.1, and ARO34442.1, respectively [10]. Capsid proteins are ideal for vaccines because they are highly immunogenic, capable of eliciting robust immune responses, and often represent conserved, structurally stable components essential for virus assembly and infectivity [11].

### Physiochemical properties and antigenicity DENV1-4

Antigenicity of each selected protein was predicted using the VaxiJen v2.0 tool with a default threshold value of 0.4, specifically tailored for viral proteins [12]. Allergenicity and toxicity evaluations were performed through AllerTOP [13] and ToxinPred platforms [14], respectively. These computational resources were subsequently employed to examine the physicochemical features and to reassess the immunogenic, allergenic, and toxicological profiles of the final multi-epitope vaccine construct.

### B cell epitopes prediction

B cell epitopes from three DENV1-4 proteins were predicted using the ABCpred server [15], employing reference protein sequences with a 12-mer length and a default threshold of 0.5. The predicted linear epitopes were subsequently evaluated for their immunogenic relevance and safety profiles using VaxiJen v2.0 to assess antigenicity, AllerTOP to determine allergenicity, and ToxinPred to screen for potential toxicity.

### Cytotoxic T cell epitopes prediction

MHC class I binding epitopes were predicted using tools available at the IEDB resource (http://tools.iedb.org/mhci/), employing the Artificial Neural Network-based NetMHC (ANN) method [16]. The analysis utilized human reference alleles, specifically HLA-A, HLA-B, and HLA-E, to identify high-affinity binders. Only conserved cytotoxic T lymphocyte (CTL) epitopes comprising nine amino acids and ranking within the top 1% percentile were selected for further evaluation. These epitopes were subsequently analyzed for their immunogenic potential, allergenic properties, and toxicity profiles to ensure their suitability in vaccine design.

### Helper T cell epitopes prediction

The reference sequence of DENV1-4 proteins was analysed for MHC class II epitope prediction using the IEDB MHC-II binding tool, employing human allele reference sets including HLA-DP, HLA-DQ, and HLA-DR. The NN-align method (NetMHCII v2.2) was applied to identify candidate epitopes with a percentile rank of ≤ 10, using core and peptide lengths of 9 and 15 amino acids, respectively [17]s. Identified helper T cell epitopes were further evaluated for antigenicity, allergenicity, and toxicity through VaxiJen v2.0, AllerTOP, and ToxinPred platforms to ensure their immunological relevance and safety.

### Assembly of the multi-epitope vaccine

The final vaccine construct was designed by integrating conserved, antigenic (score > 1), non-toxic, and non-allergenic epitopes derived from both B and T lymphocyte predictions. Helper and B-cell epitopes were linked using GPGPG [18] and KK linkers [18], respectively, while cytotoxic T-cell epitopes were joined with YAA spacers to maintain structural integrity. At the N-terminal, human β-defensin 3 (Q5U7J2) [19] was incorporated as an immune-stimulatory adjuvant via an EAAAK linker, followed by the PADRE sequence to enhance immunogenic stability. Additionally, a 6×His-tag was appended for efficient purification and detection following recombinant expression.

### 2D and 3D structure prediction of the vaccine

Predicting the 2D structure of the vaccine is available free on the PSIPRED server [20]. The secondary structure played crucial roles in predicting α-helices, β-pleated sheets, and coiled structures, respectively. For the tertiary structure prediction, the vaccine sequence was submitted to the same SWISS model [21]. The obtained results were received in the form of a PDB file that was further used for vaccine sequence refinement and adaptation.

### Vaccine 3D structure refinement and validation

The tertiary structure of the vaccine construct was refined using the GalaxyWEB server (http://galaxy.seoklab.org/) [22], enhancing its structural flexibility and stability by optimizing side-chain conformations and repacking to achieve overall relaxation. This process significantly improved the model’s structural integrity and physical reliability. Validation of the refined model was performed through the Ramachandran plot via the SAVES server (https://saves.mbi.ucla.edu.com), confirming favorable backbone dihedral angles, while structural assessment using the ProSA tool (https://prosa.services.came.sbg.ac.at/prosa.php) [23] indicated minimal errors, supporting the reliability of the final construct [24].

### Determination of the solubility of the vaccine

The solubility of the designed vaccine construct was evaluated using two independent computational platforms. Initially, the Protein-Sol server (https://protein-sol.manchester.ac.uk/) [25] applied predictive algorithms to estimate solubility, benchmarking the results against *Escherichia coli* proteins [26], which exhibit an average solubility score of 0.45; values exceeding this threshold are indicative of soluble behavior. To reinforce these findings, the SOLpro server (http://scratch.proteomics.ics.uci.edu/) was also utilized, where a solubility probability of ≥ 0.5 classifies the protein as soluble, whereas lower scores suggest poor solubility. Both analyses consistently indicated favorable solubility characteristics of the vaccine construct [27].

### Disulfide bonds in vaccine

Disulfide bonds between cysteine residues are crucial for maintaining a protein’s structural integrity and enhancing its overall stability. To introduce such covalent linkages within the vaccine construct, Disulfide-by-Design 2.0 (DbD2) [28] was employed a computational platform that identifies flexible and unstable regions in protein sequences and suggests suitable amino acid substitutions to cysteine for optimal bond formation. Parameters selected for bond modeling included intra- and inter-chain linkages, along with the option to construct Cβ atoms for glycine residues. The χ3 dihedral angle was constrained to either − 87° or + 97° with a tolerance of ± 30°, while the Cα–Cβ–Sγ bond angle was fixed at 114.6° ±10°, ensuring geometrically favorable disulfide linkage predictions.

### Prediction of discontinuous B-cell epitopes

The prediction of conformational B-cell epitopes was conducted using ElliPro available through the IEDB analysis resource (http://tools.iedb.org/ellipro/) [29], which identifies both linear and discontinuous antibody-binding regions by evaluating the three-dimensional structure of the protein. The tool employs a structure-based algorithm, and the analysis was performed using its standard configuration, with a minimum score threshold of 0.5 and a maximum distance parameter of 6 Å for epitope definition.

### Molecular docking of the vaccine protein with TLRs

The interaction patterns between the designed vaccine construct and Toll-like receptors (TLRs) were investigated through molecular docking to assess their immunological relevance [30]. TLR4 and TLR8 were selected due to their critical functions in pathogen detection, immune signaling, dendritic cell activation, and T helper cell modulation [31]. TLR4, in particular, is widely recognized for identifying diverse microbial signatures, partly attributed to its cooperative activity with other TLRs such as TLR1 and TLR6. We selected TLR4 and TLR8 for docking studies due to their pivotal roles in antiviral immunity. TLR4 is known to recognize diverse microbial antigens and initiate pro-inflammatory signaling that enhances adaptive immune responses, while TLR8 specifically detects single-stranded RNA viruses like Dengue, activating dendritic cells and T helper cells. Docking the MEV-DV construct with these receptors allowed us to evaluate its potential to effectively stimulate innate immune pathways, thereby supporting strong and coordinated humoral and cellular responses. To simulate receptor-ligand engagement, ClusPro 2.0 [32, 33] was employed to generate multiple docking conformations, ranked based on cluster scores reflecting model stability. Subsequently, refinement of the most favorable complexes was achieved using HADDOCK 2.4 [34], which optimized interfacial side-chain orientations and minimized the energy of the docked structures, enhancing the structural reliability of vaccine–TLR interactions.

### Molecular dynamics simulation

Molecular dynamics simulations were conducted using GROMACS v2022.3 [35] to investigate the stability and structural behavior of the post-docking vaccine–TLR complex. The Amber99SB-ILDN force field was selected for its suitability in modeling protein interactions, allowing the construction of topology and coordinate files for the system. Solvation was achieved with TIP3P water molecules, and the system’s neutrality was ensured by adding appropriate counter-ions. Energy minimization proceeded iteratively to reach a stable conformation. To mimic physiological conditions, equilibration phases were executed under constant volume and temperature (NVT) for 400 ps and under constant pressure and temperature (NPT) for 1 ns, maintaining 310 K and 1 atm using the Berendsen thermostat and Parinello-Rahman barostat. The 50 ns production run enabled the extraction of critical dynamic properties, including root mean square deviation, root mean square fluctuation, radius of gyration, and hydrogen bond patterns. Constraints on hydrogen bonding were applied using the LINCS algorithm, and long-range electrostatic interactions were efficiently managed through the Particle-Mesh Ewald method [36].

### Immune simulation

Using a rapid Position Specific Scoring Matrix (PSSM)-based approach, the C-ImmSim server (https://kraken.iac.rm.cnr.it/C-IMMSIM/index.php) [37] was employed to simulate the anticipated human immune response. The simulation encompassed 1050 time steps, equivalent to 350 days, with three vaccine doses delivered at steps 1, 86, and 164 each spaced four weeks apart administering 1000 units per dose [38]. All remaining simulation parameters were maintained at their default settings.

### Vaccine design, peptide synthesis, and characterization

The multi-epitope vaccine construct against Dengue Virus (MEV-DV) was designed using a computational immunoinformatics approach integrating B-cell, helper T-cell, and cytotoxic T-cell epitopes from the envelope (E), NS1, and NS3 proteins. A universal pan-allelic T-cell epitope (PADRE sequence) and a 6xHis tag were incorporated at the N- and C-termini, respectively, to enhance immunogenicity and facilitate downstream characterization [39]. The finalized construct, comprising 40 amino acids, was codon-optimized for *Mus musculus* and synthesized commercially by GenScript Biotech^®^ (Singapore).

The lyophilized peptide was analyzed for physicochemical parameters using ExPASy ProtParam. The predicted molecular weight was 4.44 kDa, theoretical isoelectric point (pI) was 9.78, and the overall charge at physiological pH was + 2.96, indicating a mildly basic and moderately hydrophilic nature suitable for aqueous formulation [40].

### Peptide reconstitution and homogenization

Three lyophilized peptide vials were received, labeled MEV-DV-P1, P2, and P3, and stored at − 80 °C until use. Each vial was reconstituted using ultrapure Milli-Q water to achieve a final concentration of 1 µg/µL. One-third of the total peptide from each vial was transferred to sterile 15 mL Falcon tubes and mixed individually using a vortex mixer [41]. The reconstituted solutions were pooled and homogenized using a Comecta Ivymen^®^ Ultrasonic Homogenizer (Barcelona, Spain) with three 10 s pulses at 20 kHz to ensure complete dispersion of peptide aggregates.

The homogenized mixture was stored at 4 °C overnight to assess solubility stability. No precipitate formation was observed the next day, confirming adequate peptide solubilization. Aliquots of 750 µL were dispensed into 1.5 mL cryovials and stored at − 80 °C as stock solutions for subsequent vaccine formulation.

### Preparation of alum-based adjuvant and vaccine formulation

A 20% (w/v) alum adjuvant was prepared by dissolving 10 g of potassium aluminum sulfate (KAl(SO₄)₂·12 H₂O) in 50 mL sterile phosphate-buffered saline (PBS, pH 7.4). The solution was stirred continuously at 60 °C until fully dissolved and then cooled to room temperature under aseptic conditions [42]. For vaccine formulation, 25 µg of MEV-DV peptide was mixed with 4 mg of alum adjuvant in a sterile microtube by adding 750 µL of peptide stock to 600 µL of 20% alum solution. The mixture was subjected to short ultrasonication bursts (3 × 10 s at 20 kHz) to ensure proper adsorption of the peptide antigen onto the alum particles. The prepared formulation was kept at 4 °C until administration. A control formulation containing alum only and another containing sterile PBS were also prepared following the same protocol.

### Experimental animals

All animal procedures were conducted in accordance with the ARRIVE guidelines and the NIH Guide for the Care and Use of Laboratory Animals (NIH, 2011). A total of 63 healthy, 8-week-old albino mice (*Mus musculus*, VRI-PA strain) were obtained from the Veterinary Research Institute (VRI), Peshawar. Mice were housed in standard pathogen-free cages at 22 ± 2 °C, with a 12 h light/dark cycle and ad libitum access to food and water. Animals were acclimatized for one week prior to immunization [43].

### Immunization protocol

The mice were randomly divided into three groups (*n* = 21 per group):

1. *Group 1*: Received 0.1 mL of alum-adjuvanted MEV-DV formulation (experimental vaccine).
2. *Group 2*: Received 0.1 mL of commercial inactivated Dengue vaccine (reference control).
3. *Group 3*: Received 0.1 mL of sterile PBS (negative control).

Each mouse was injected subcutaneously in the brachial region (near the armpit). The primary (prime) dose was administered on day 0, followed by a booster dose on day 14 using the same route and volume. All animals were closely monitored for local reactions or behavioral changes throughout the study.

### Blood collection and serum preparation

Blood samples were collected from the retro-orbital plexus using sterile 1 mL syringes at days 0, 7, 14, 21, 28, 35, and 42 post-vaccination. Samples were allowed to clot at 37 °C for 1 h and centrifuged at 3000 rpm for 10 min. The resulting serum was separated and stored at 4 °C for immediate analysis or at − 20 °C for long-term storage.

### Hemagglutination Inhibition (HI) Assay

Serum antibody titers were quantified using the hemagglutination inhibition (HI) assay, following standard protocols with minor modifications. Briefly, 25 µL of serially diluted serum samples were mixed with an equal volume of 4 HA units of Dengue Virus antigen in V-bottom microtiter plates and incubated at room temperature for 30 min. Subsequently, 25 µL of 0.5% chicken red blood cell suspension was added to each well. Plates were gently tapped to mix and observed for agglutination after 30 min.

The HI titer was defined as the highest serum dilution that completely inhibited hemagglutination. Mean titers and standard deviations (SD) were calculated for each group at every time point.

### Statistical analysis

All data were expressed as mean ± standard deviation (SD). Statistical analysis was conducted using GraphPad Prism^®^ (version 10.0). Differences between the vaccinated and control groups were evaluated using a two-tailed independent t-test. A *p*-value < 0.05 was considered statistically significant.

## Results

### Retrieval of immunogenic capsid proteins

By utilizing the NCBI database the three-capsid protein sequence are downloaded by accession no. NP_059433.1, NP_739591.2, A0A217EIR2, ARO34439.1, ARO34441.1, and ARO34442.1, respectively. The antigenicity, allergenicity and toxicity of these proteins are assessed shows in Table 1.

**Table 1:** Shows the antigenicity, allergenicity and toxicity of capsid proteins

Accession ID	Antigenicity	Allergenicity	Toxicity
NP_059433.1	0.4553	Non	Non
NP_739591.2	0.4573	Non	Non
A0A217EIR2	0.4663	Non	Non
ARO34439.1	0.4473	Non	Non
ARO34441.1	0.4563	Non	Non
ARO34442.1	0.4513	Non	Non

### Identification of T cell epitopes

T-cell epitopes, composed of short peptides, can activate immune responses by binding to specialized receptors on cytotoxic T-cells (CTLs). These receptors recognize antigens presented on the surface of infected cells, forming a specific complex. Using the IEDB server, conserved sequences from three proteins were identified to design T-cell epitopes for MHC class I and II. Selected epitopes demonstrated high binding affinity, broad HLA compatibility, and favorable properties, including high antigenicity, non-allergenicity, non-toxicity, surface localization, and complete conservancy across DENV genotypes (Table S1). Three 9-mer CTL epitopes with conserved sequences were identified as potential vaccine targets due to their ability to engage CD8 + T-cells via HLA class I binding, promoting cellular immunity. Similarly, three 15-mer helper T-cell (HTL) epitopes with cytokine-inducing properties were chosen for their ability to bind HLA class II, stimulating both humoral and cellular responses through CD4 + T-cells (Table S2).

### B cell epitopes prediction

B-cell epitopes are pivotal in vaccine strategies designed to prevent specific diseases. For effective immune activation, these epitopes must exhibit antigenicity and be exposed on the surface. The epitopes derived from capsid proteins were selected through rigorous evaluation of their antigenic potential, allergenic risks, and toxicity profiles. Notably, all chosen epitopes are devoid of allergenic and toxic properties, highlighting their suitability as vaccine candidates (Table S3, Figure S1, S2 and S3). By interacting with B-cell receptors, these epitopes initiate humoral or cellular immune responses, which are fundamental to the development and effectiveness of vaccines. The full list of Alleles are present in Table S4.

### Multi-epitope vaccine design

**Fig. 3:**
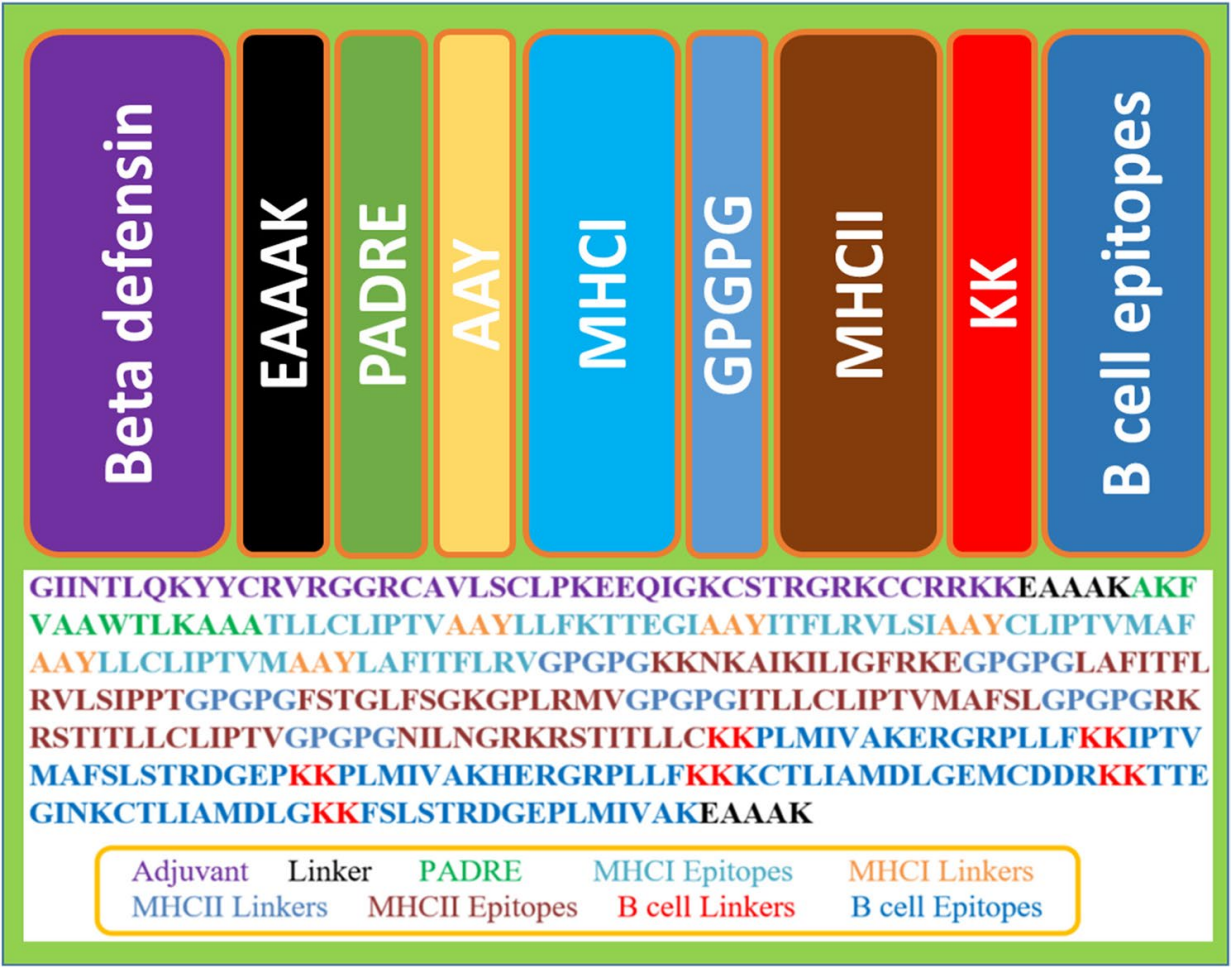
Presents the vaccine construct with respective adjuvants, linkers and epitopes

Adjuvants and linkers are essential components for optimizing vaccine performance by improving immune response, structural integrity, and overall efficacy. In this study, a vaccine construct was engineered using a β-defensin-based adjuvant, integrated with a PADRE sequence and strategically selected linkers EAAAK, AAY, GPGPG, and KK to effectively join the adjuvant with CTL, HTL, and linear B-cell epitopes, respectively (Fig. 3). Comprehensive in silico evaluations confirmed the designed construct to be safe, exhibiting strong antigenic properties while lacking toxic or allergenic potential.

### Physiochemical properties

The designed vaccines demonstrated a high antigenicity score of 0.8559, supporting their suitability for subsequent in vivo evaluation. Allergenicity analysis was performed via a comprehensive method combining SVM-based modeling, IgE epitope identification, alignment with allergenic protein sequences (ARPs BLAST), and motif scanning (MAST) using the AlgPred platform. The support vector machine prediction, based on amino acid composition, applied a decision threshold of -0.4 to distinguish potential allergens.

The physicochemical properties of the multi-epitope vaccine (MEV) were evaluated using the ProtParam web tool, revealing favorable attributes for its potential application. Comprising 364 amino acids and 5703 atoms, the construct has a molecular formula of C_1781_H_2961_N_479_O_456_S_26_ and a molecular mass of approximately 39 kDa. It exhibited a theoretical isoelectric point (pI) of 10.17, with 59 positively and 18 negatively charged residues, indicating a net positive charge. The calculated instability index of 27.27 suggests the protein is stable, while an aliphatic index of 101.37 reflects strong thermostability. Additionally, the GRAVY score of -0.271 implies a hydrophilic profile, enhancing its potential solubility. A summary of these parameters is presented in Table 2.

**Table 2:** Results of physicochemical properties analysis

Physical and Chemical Properties	Result
Number of amino acids	364
Molecular weight	39214.60
Theoretical-pI	10.17
Total number of negatively charged residues (Asp + Glu)	18
Total number of positively charged residues (Arg + Lys)	59
Formula	C_1781_H_2961_N_479_O_456_S_26_
Total number of atoms	5703
Instability-index	27.27
Aliphatic-index	101.37
Grand average of hydropathicity (GRAVY)	-0.271

The vaccine construct, with a GRAVY score of -0.271, demonstrates a hydrophilic profile supported by its amino acid composition, comprising 18 negatively and 59 positively charged residues. This physicochemical characterization confirms its affinity for aqueous environments, aligning with the updated interpretation to reflect accurate computational findings.

### 2D and 3D model prediction, refinement and validation

**Fig. 4:**
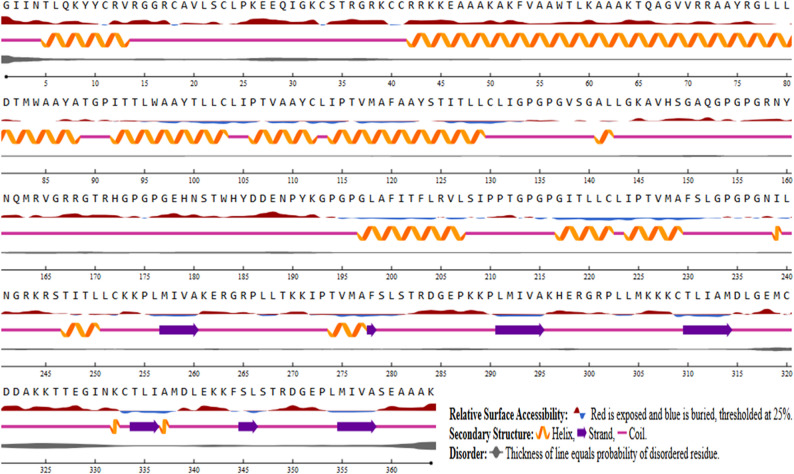
Shows the secondary structure of vaccine construct and alpha helix, strand and coil

**Fig. 5:**
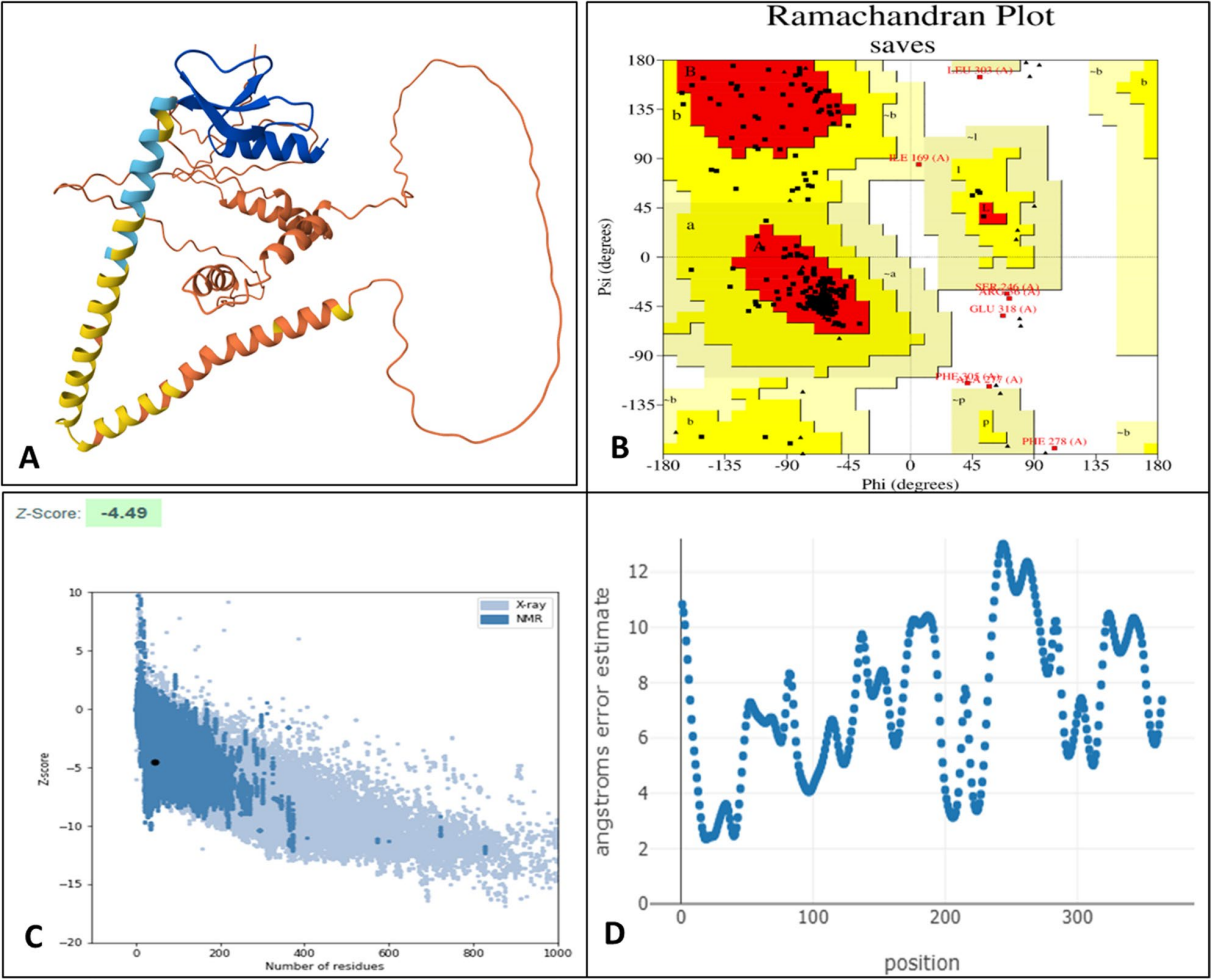
In the tertiary structure modeling, refinement, and quality validation of the vaccine construct: (**A**) Shows the 3 dimensional structure of Vaccine construct (**B**) Ramachandran plot analysis indicates that 99.02% of residues are in the favored region, with 1.9% in the allowed region and 0.1% in the disallowed region (**C**) The ProSA-web plot affirms the acceptable quality of the vaccine model (**D**) Shows the error estimation of Angstroms

The secondary structure of the vaccine consists of α-helix, β-sheets, and random coils, as depicted in the Fig. 4 and residues properties of absolute deviation from mean chi_1 value, omega torsion, zeta torsion and G-factors are shown in Figure S4. To predict the three-dimensional structure in the absence of a suitable homologous template, AlphaFold 3.0 was utilized, yielding a model with a C-score of − 3.43, indicative of moderate confidence. Refinement via GalaxyRefine generated five optimized variants, with a TM-score of 0.34 ± 0.11 and an RMSD of 14.2 ± 3.8 Å. Structural validation through the SAVES server its Overall ERRAT Quality Factor 99.7167 and Ramachandran plot revealed 97.6% of residues positioned in favored regions, supporting stereochemical soundness. Additionally, the model’s overall reliability was confirmed with a Z-score of − 5.26 and an ERRAT quality factor, demonstrating its suitability for downstream analyses (Fig. 5).

### Discontinuous B-cell epitope

**Fig. 6:**
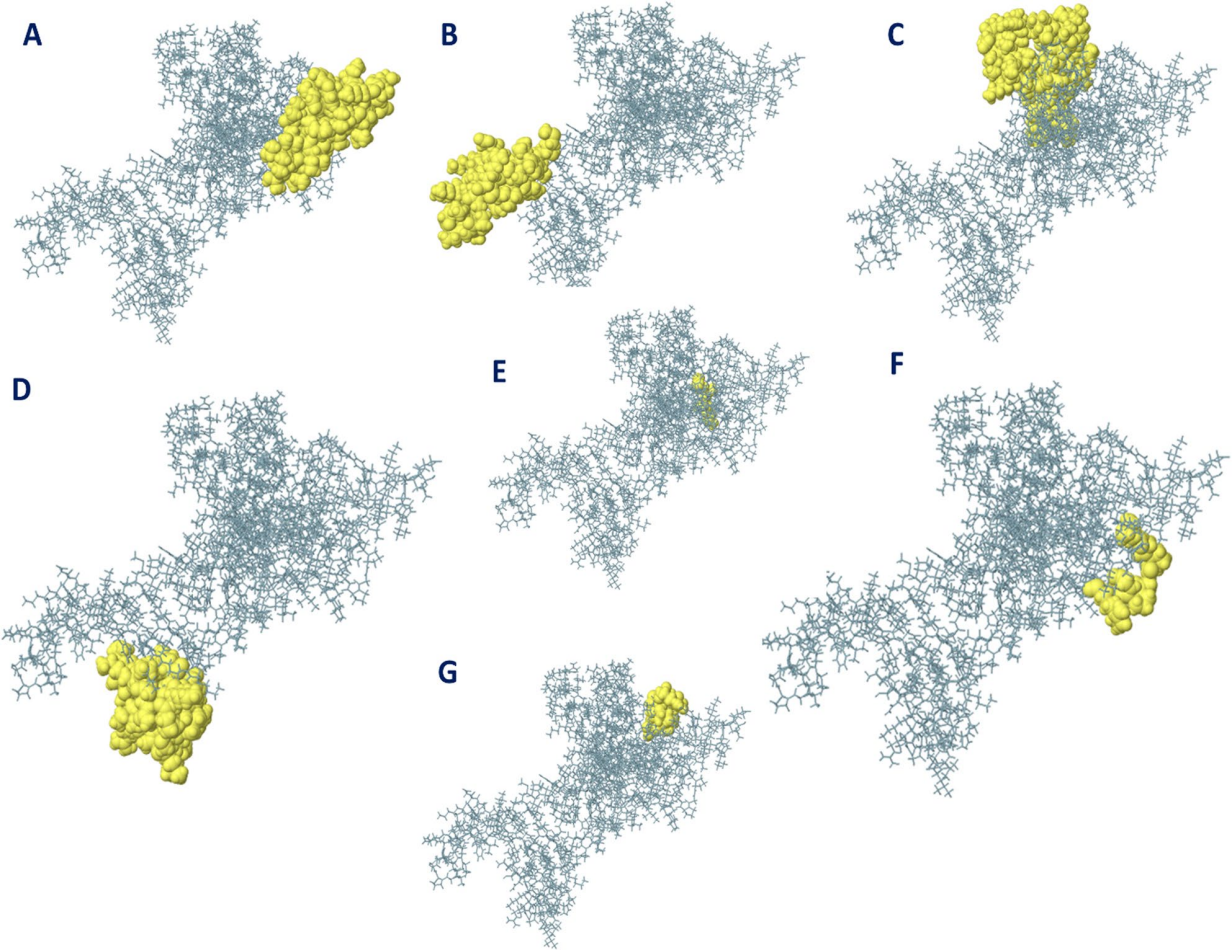
Conformational B-cell epitopes prediction in MEV. The light-yellow spheres display residues containing regions predicted conformational B-cell epitope regions on the MEV structure (gray-blue). (A) Front view of the MEV showing surface-exposed epitope residues. (B) Side view highlighting epitope distribution on the upper region. (C) Top view demonstrating clustered epitope residues. (D) Lower-side orientation showing peripheral epitope localization. (E) Central surface view illustrating partially exposed epitope regions. (F) Opposite-side orientation displaying additional conformational epitopes. (G) Rear view showing widespread distribution of predicted B-cell epitope residues across the MEV surface

The vaccine construct predicts four discontinuous epitopes, with residue sizes ranging from 4 to 10 and scores between 0.54 and 0.811, as detailed in Table S4 and Fig. 6.

### Determination of the solubility of the vaccine

**Fig. 7:**
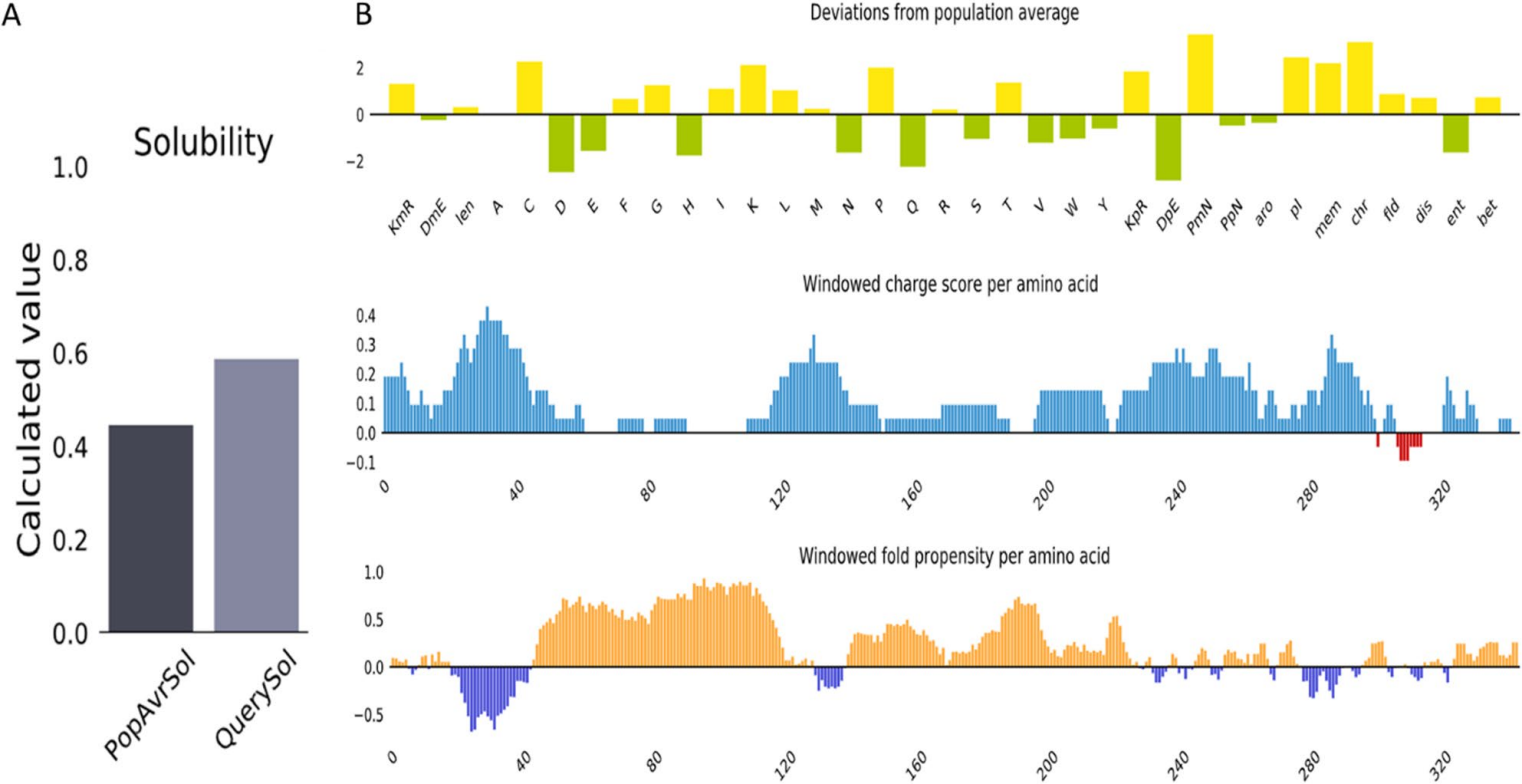
Protein-sol analysis of the vaccine construct. (**A**) Predicted solubility of the vaccine protein (QuerySol) compared to the population average (PopAvrSol). (**B**) Sequence-based features showing amino acid composition deviation, charge distribution, and fold propensity, indicating good solubility and structural stability

The solubility of the designed multi-epitope vaccine protein was predicted using the Protein-sol server, which evaluates protein solubility based on sequence features and comparison to a dataset of experimentally characterized proteins. As shown in the Fig. 7, the solubility score of the vaccine construct (QuerySol ≈ 0.6) was higher than the average solubility of the reference protein dataset (PopAvrSol ≈ 0.45), indicating a favorable solubility profile. The amino acid composition analysis revealed moderate deviations from the population average, with certain residues slightly over- or under-represented, suggesting no significant detrimental effect on solubility. The windowed charge score per amino acid demonstrated consistent positive charge distribution across the sequence, with only a minor dip observed near residue 320, implying that the construct maintains a favorable electrostatic profile. Additionally, the windowed fold propensity analysis indicated a strong tendency of the vaccine protein to adopt a stable folded conformation, with most regions showing high fold propensity and only a few isolated segments displaying lower values. Collectively, these results suggest that the vaccine protein is predicted to be well-folded and soluble, making it suitable for downstream expression and purification processes.

### Disulfide engineering of MEV

**Fig. 8:**
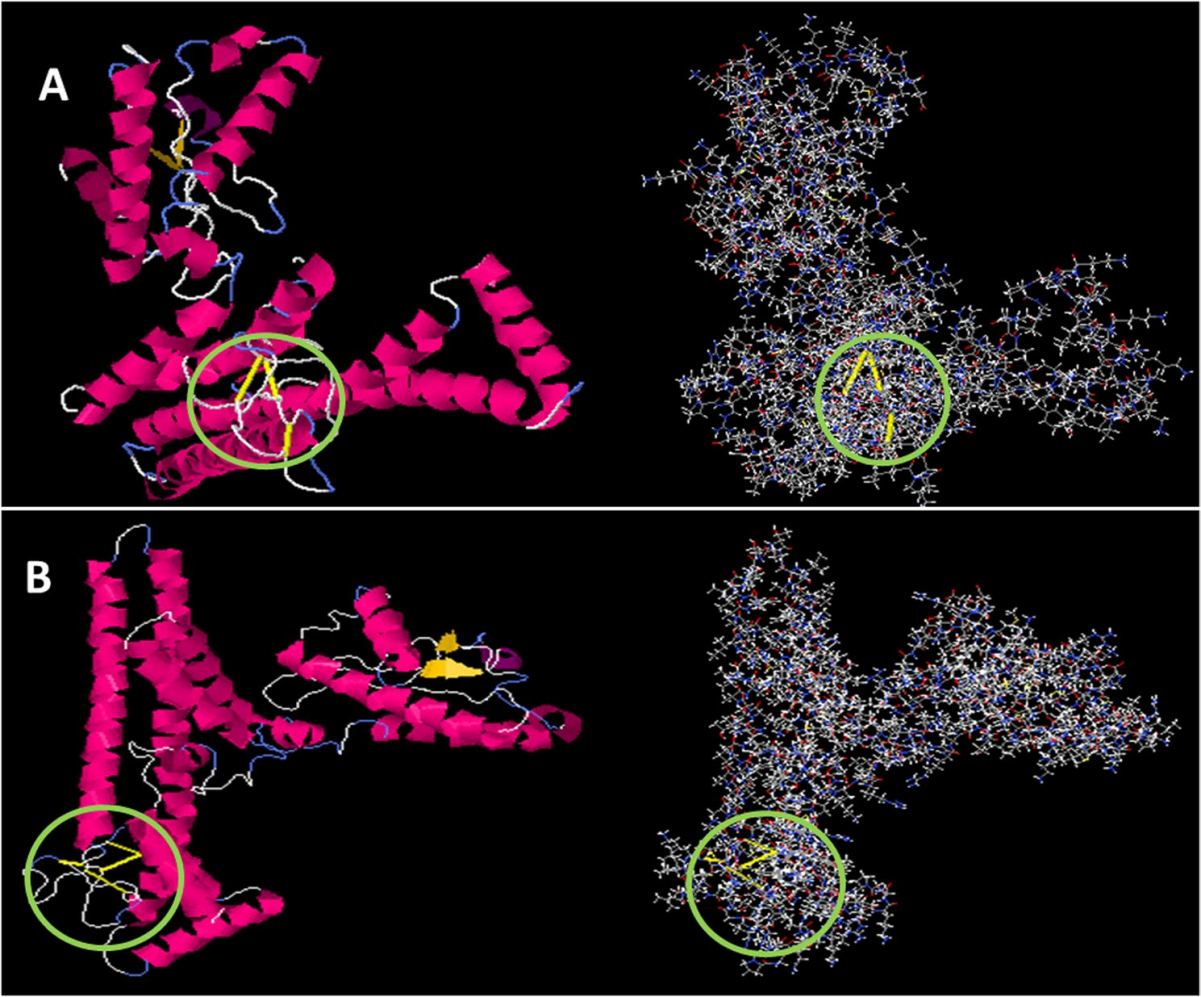
**(A)** Shows original and **(B)** shows mutant. The yellow shows the disulphide bonds in the vaccine

The Disulfide by Design 2 server was employed to predict disulfide bond-forming sites within the MEV protein by analyzing amino acid pairs with favorable bond energies. Four residue pairs were identified with energies below 3.15 kcal/mol, suggesting potential for stable disulfide linkages: CYS11–GLY31, CYS33–CYS40, GLU27–CYS41, and GLY16–ARG42. These candidate interactions indicate structurally feasible sites for disulfide engineering. Figure 8 illustrates the comparison between native and modified MEV conformations, highlighting the aforementioned residue pairs as potential sites for enhancing protein stability.

### Molecular docking

**Fig. 9:**
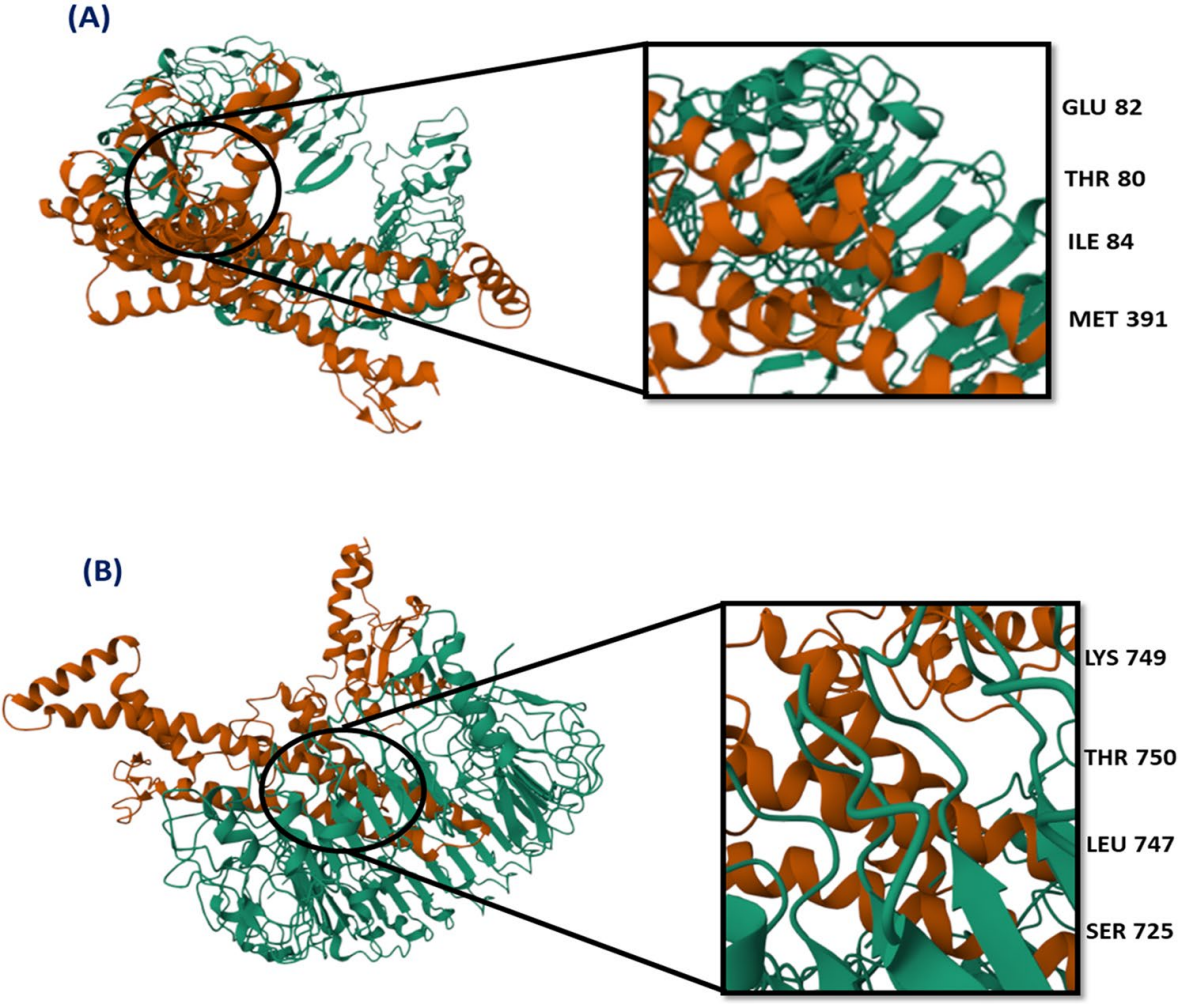
Docked structure of TLRs with MEV (**A**) shows the TLR4 receptor interaction with MEV (**B**) shows the TLR8 receptor interaction with MEV, both also represents attached labelled amino acids

Molecular docking analysis was conducted to evaluate the interaction between the MEV and immune receptors TLR4 (PDB ID: 3FXI) and TLR8 (PDB ID: 3W3M) using the ClusPro 2.0 server. From the generated models, the ten highest-ranking docked complexes were shortlisted for each receptor, with the most favorable conformation selected based on energy parameters and docking scores (Table 3; Fig. 9). Notably, the MEV-TLR8 complex demonstrated the strongest predicted affinity, reflected by its minimum binding energy of -1366.2 kJ/mol and a center score of -1038.0.

**Table 3:** Presents the vaccine construct docking score and energies with receptors

Docking	MEV-TLR4	MEV-TLR8
Cluster	1	3
Member	41	37
Score	-971	-1038.0
Energy	-1336.4	-1366.2

### Molecular dynamic simulation

**Fig. 10:**
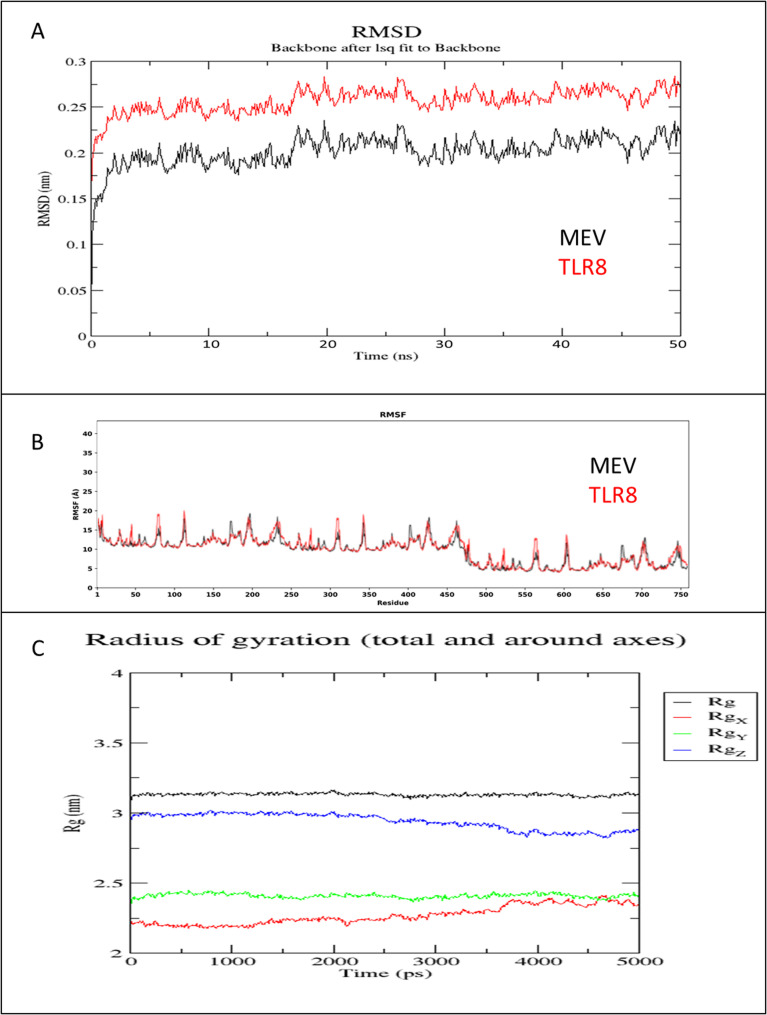
MD simulation analysis of the MEV–TLR8 complex over a 50 ns period. (**A**) RMSD plot showing structural stability of MEV (black) and TLR8 (red), indicating early equilibrium and minimal deviations. (**B**) RMSF plot representing residue-wise flexibility, with higher fluctuations observed in loop regions, particularly in TLR8. (**C**) Radius of gyration (Rg) plot demonstrating overall compactness and structural integrity of the complex, with stable Rg values throughout the simulation. These results support the conformational stability of the vaccine–receptor complex

The molecular dynamics (MD) simulation of the MEV–TLR8 complex was conducted over a 50 ns period to assess the structural stability and compactness of the vaccine-receptor interaction, which is critical for evaluating the potential of MEV (multi-epitope vaccine) as an effective immunogen. As shown in Fig. 10A, the RMSD (Root Mean Square Deviation) plot indicates that both MEV (black line) and TLR8 (red line) achieved structural equilibrium early in the simulation and remained stable throughout the 50 ns duration. MEV exhibited a slightly lower RMSD (\~0.20 nm) compared to TLR8 (\~0.25–0.30 nm), suggesting that the vaccine component retains a stable and consistent backbone conformation during the simulation. Figure 10B presents the RMSF (Root Mean Square Fluctuation) data, which measures the flexibility of individual amino acid residues. The results show moderate fluctuations, mostly in loop and surface-exposed regions, particularly in TLR8, indicating some dynamic motion without compromising the overall structural integrity of the complex. These flexible regions may play roles in antigen recognition or immune signaling. Figure 10C illustrates the radius of gyration (Rg), which remained relatively stable over the course of 5000 ps, with minimal variations around the X, Y, and Z axes, further confirming that the complex maintained a compact and rigid structure throughout the simulation. Together, these results demonstrate that the MEV–TLR8 complex exhibits significant structural stability, an essential feature for successful vaccine interaction with immune receptors, thereby supporting the vaccine construct’s potential as a promising candidate for further immunological studies and development.

### Immune simulation

Immune simulation serves as a critical component in rational vaccine design, offering a computational framework to predict and analyze immune dynamics following antigen exposure. These simulations incorporate detailed interactions between innate and adaptive immune compartments, enabling the characterization of humoral and cellular responses essential for long-term immunity. Innate immunity, comprising macrophages, dendritic cells, and other antigen-presenting cells, initiates immune defense by detecting pathogens, processing antigens, and secreting cytokines that modulate the adaptive response. Understanding these interactions through simulation helps identify correlates of protection and optimize vaccine parameters, including dosage, timing, and adjuvant selection.

**Fig. 11:**
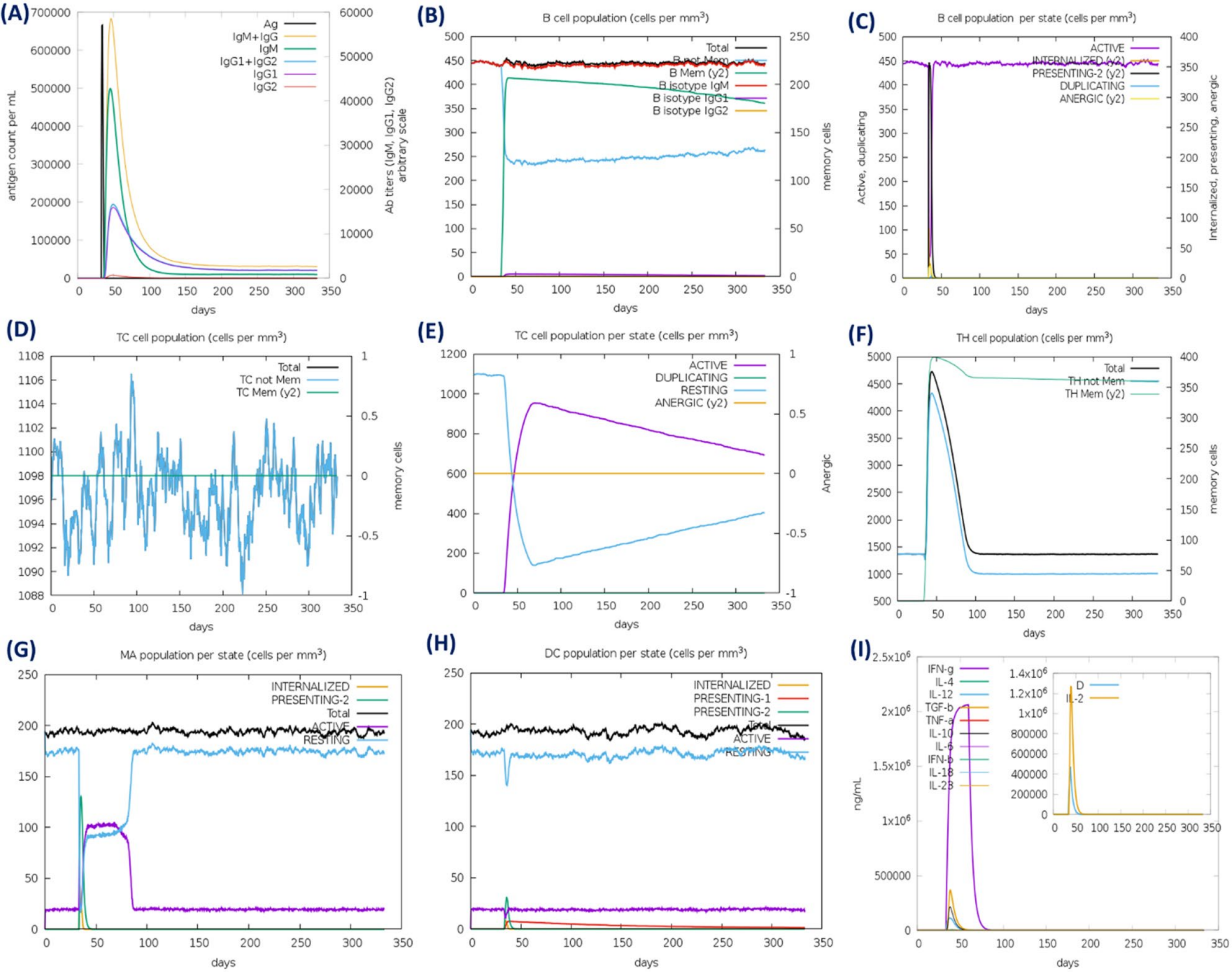
Simulated immune response following antigen exposure over a 350-day period. (**A**) Antigen clearance and dynamics of immunoglobulin isotypes (IgM, IgG1–4). (**B**) Total and memory B cell populations. (**C**) B cell states: active, duplicating, anergic, and presenting. (**D**) Cytotoxic T cell population with memory cell dynamics. (**E**) TC cell states over time. (**F**) TH population and memory TH cells. (**G**) Macrophage population distribution across functional states. (**H**) Dendritic cell population by activation and presentation status. (**I**) Cytokine concentration dynamics including IFN-γ, IL-2, IL-12, and others. The inset highlights IL-2 and TGF-β profiles. Data represent a coordinated cellular and humoral immune response following simulated vaccination

The presented simulation results depict a coordinated immune response over a period of 350 days. Figure 11A the antigen (Ag) concentration peaks rapidly and declines as immunoglobulin isotypes (IgM, IgG1, IgG2, IgG3, IgG4) are produced, with IgG1 showing a pronounced and sustained increase, indicative of class switching and maturation of the humoral response. Figure 11B reveals an early expansion and stabilization of total B cell populations, with a gradual increase in memory B cells. Figure 11C delineates B cell activation states, where a transient rise in duplicating and presenting B cells is followed by a predominance of active and anergic cells. Cytotoxic T cell (TC) populations (Fig. 11D, E) remain stable in total number, with a marked rise in memory TC cells and dynamic changes in activation status, showing an early dominance of active cells transitioning into duplicating and resting phases. Helper T cells (TH) in Fig. 11F follow a similar trend, characterized by a peak in total TH cells and sustained presence of memory TH cells. Macrophage populations Fig. 11G exhibit early internalization and antigen presentation, followed by a resting state. Dendritic cells Fig. 11H demonstrate rapid activation and antigen presentation shortly after antigen exposure. Finally, Fig. 11I illustrates a sharp, transient increase in key cytokines (e.g., IFN-γ, IL-2, IL-12), which corresponds temporally with early antigen clearance and immune activation. These results collectively represent a robust, multi-phase immune response consistent with effective immunization, highlighting the utility of simulation in predicting vaccine-induced immunity.

### Immunogenicity of the Multi-Epitope Vaccine (MEV-DV) in mice

**Fig. 12:**
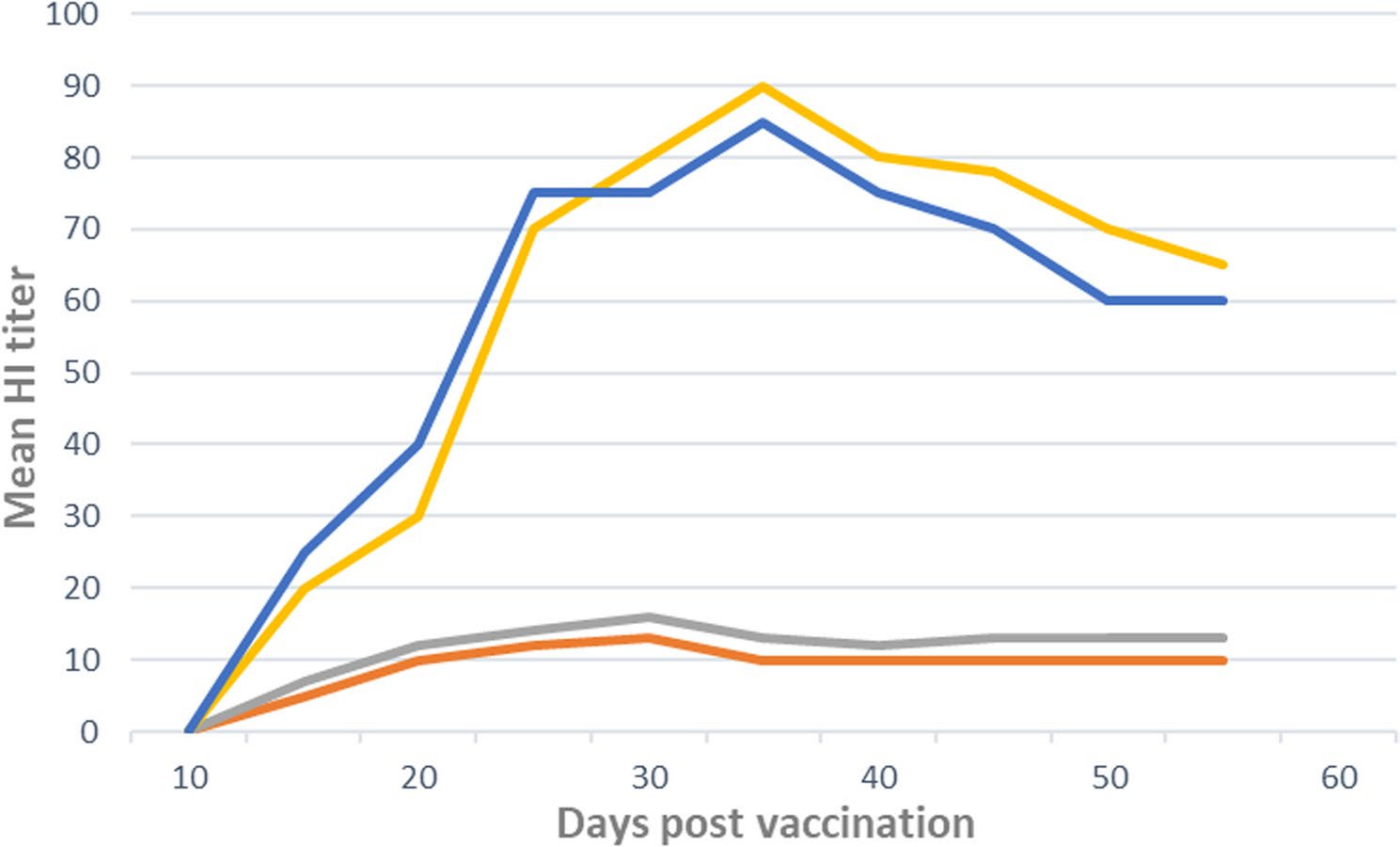
Comparative immune response of albino mice immunized with the MEV-DV multi-epitope vaccine (Yellow line), reference vaccine (blue line), and PBS control (orange line). The vertical axis represents HI titers, and the horizontal axis represents post-vaccination days. Each label indicates the day and mean titer corresponding to that time point

To evaluate the immune response induced by the designed multi-epitope vaccine (MEV-DV), albino mice were immunized with either the alum-adjuvant MEV-DV formulation (Group 1), a commercial inactivated dengue vaccine as reference (Group 2), or sterile PBS as the negative control (Group 3). Serum samples were collected from all groups at 0, 7, 14, 21, 28, 35, and 42 days post-vaccination to measure the antibody titers using the hemagglutination inhibition (HI) assay. No significant immune response was observed in the PBS control group throughout the study period, confirming the specificity of the vaccine-induced antibodies. The results are summarized in Table 4 and illustrated in Fig. 12.

**Table 4:** Hemagglutination inhibition (HI) titers of serum samples from albino mice immunized with MEV-DV, reference vaccine, and PBS control at various days post-vaccination

Day	Group 1 (MEV-DV)			Group 2 (Reference Vaccine)			Group 3 (PBS Control)		
	Sample 1	Sample 2	Sample 3	Sample 1	Sample 2	Sample 3	Sample 1	Sample 2	Sample 3
0	0	0	0	4	4	4	4	4	4
7	32	16	32	16	32	16	8	8	4
14	128	128	64	64	64	64	4	2	4
21	128	128	128	128	256	128	4	8	4
28	64	128	64	256	128	128	2	8	8
35	32	32	64	128	256	128	4	8	4
42	16	16	32	128	128	128	8	4	4
**Day**	**Mean HI Titer (MEV-DV)**	**Mean ± SD**	**Mean HI Titer (Reference Vaccine)**	**Mean ± SD**	**Mean HI Titer (PBS)**	**Mean ± SD**			
0	0.00	–	4.00	–	4.00	0.00			
7	26.67	7.64	21.33	8.93	6.67	1.25			
14	106.67	37.93	64.00	0.00	3.33	1.25			
21	128.00	0.00	170.67	63.54	5.33	2.31			
28	85.33	32.00	170.67	67.45	6.00	3.46			
35	42.67	15.97	170.67	63.54	5.33	1.15			
42	21.33	7.64	128.00	0.00	5.33	1.15			

Bold values indicate statistical significance (p < 0.05)

### Comparative antibody response and statistical analysis

A comparative evaluation using an independent *t*-test (two-tailed) revealed no statistically significant differences between the MEV-DV and reference vaccine groups at any sampling time point (*p* > 0.05). Although both vaccines elicited similar magnitudes of humoral response, the MEV-DV vaccine demonstrated comparable antibody kinetics with a slightly earlier onset and stable titers post-booster (Table 5).

**Table 5:** Comparison of mean HI titers between MEV-DV and reference vaccine groups in albino mice

Day	MEV-DV Group (Mean ± SD)	Reference Vaccine Group (Mean ± SD)	*p*-value
0	0.00 ± 0.00	4.00 ± 0.00	0.195
7	26.67 ± 7.64	21.33 ± 8.93	0.482
14	106.67 ± 37.93	64.00 ± 0.00	0.127
21	128.00 ± 0.00	170.67 ± 63.54	0.263
28	85.33 ± 32.00	170.67 ± 67.45	0.136
35	42.67 ± 15.97	170.67 ± 63.54	0.082
42	21.33 ± 7.64	128.00 ± 0.00	0.057

### Graphical analysis of humoral response

The pattern of antibody titers in mice over 42 days is illustrated in Fig. 12. The MEV-DV group (orange line) displayed a sharp rise after day 7, reaching the peak titer at day 21, which was sustained until day 28. The reference vaccine group (blue line) showed a similar response profile but with slightly higher overall titers. The PBS control (green line) remained at baseline levels throughout the study.

This result demonstrates that the computationally designed MEV-DV construct is capable of eliciting a potent and sustained humoral immune response in mice, comparable to the commercial reference vaccine.

The MEV-DV vaccine induced a strong and consistent antibody response, with titers peaking by day 21. The kinetics of antibody production were comparable to those of the reference vaccine, suggesting successful immune activation. No adverse reactions were observed in any mice, confirming the safety and tolerability of the vaccine formulation. The statistically non-significant difference between MEV-DV and the reference vaccine indicates immunological equivalence, validating the efficacy of the computationally designed construct.

## Discussion

Dengue virus (DENV) continues to represent a significant global public health challenge due to the co-circulation of four antigenically distinct serotypes (DENV1–4) and the complex immunopathological phenomenon of antibody-dependent enhancement (ADE) [44]. Despite decades of vaccine research, the development of a universally protective and safe dengue vaccine remains elusive, largely because balanced and durable immunity must be achieved against all serotypes simultaneously. In this study, we employed an integrated reverse vaccinology, immunoinformatics, and experimental validation strategy to design and evaluate a conserved capsid-based multi-epitope vaccine (MEV-DV) targeting DENV1–4. Our findings demonstrate that this rationally designed construct possesses favorable structural, immunological, and safety characteristics and is capable of inducing a robust humoral immune response in a murine model [6, 45].

The selection of the capsid protein as a vaccine target represents a strategic departure from conventional dengue vaccine approaches that primarily focus on envelope (E) and NS1 proteins. Although E protein-based vaccines are highly immunogenic, they are also subject to substantial sequence variability among serotypes and are strongly implicated in ADE through the generation of cross-reactive but non-neutralizing antibodies. In contrast, the capsid protein is highly conserved across DENV1–4 and plays a critical role in viral assembly and replication [46, 47]. Previous studies have shown that capsid-derived epitopes can effectively stimulate T-cell responses and contribute to viral clearance. By focusing on conserved capsid epitopes, the present study aimed to minimize serotype bias while maximizing cross-serotype immune coverage and safety.

Using comprehensive immunoinformatics pipelines, we identified and screened B-cell, cytotoxic T lymphocyte (CTL), and helper T lymphocyte (HTL) epitopes based on antigenicity, conservancy, non-allergenicity, and non-toxicity [48, 49]. The incorporation of a β-defensin adjuvant and PADRE sequence further enhanced the immunogenic potential of the construct by promoting innate immune activation and broad HLA coverage [50]. The use of appropriate linkers (EAAAK, AAY, GPGPG, and KK) ensured proper epitope separation and structural flexibility, facilitating optimal antigen processing and presentation. Physicochemical analysis revealed that the MEV-DV construct is stable, soluble, and hydrophilic, properties that are essential for recombinant expression and formulation. Structural modeling and validation confirmed the conformational integrity of the vaccine construct, with the majority of residues located in favored regions of the Ramachandran plot and an acceptable ProSA Z-score. Disulfide engineering further enhanced structural stability, which is particularly important for maintaining epitope conformation and prolonging antigen persistence in vivo. These results support the structural feasibility of the designed vaccine and its suitability for downstream experimental evaluation [51].

A key aspect of vaccine efficacy lies in the ability to activate innate immune receptors and initiate adaptive immune responses. Molecular docking analyses revealed strong binding affinities between MEV-DV and Toll-like receptors TLR4 and TLR8, with particularly stable interactions observed with TLR8. TLR8 is known to recognize single-stranded RNA viruses and plays a pivotal role in dendritic cell activation and cytokine secretion, thereby bridging innate and adaptive immunity [52]. Molecular dynamics simulations further demonstrated that the MEV–TLR8 complex remained stable over a 50 ns simulation period, indicating sustained receptor engagement under physiological conditions. These findings suggest that MEV-DV can effectively stimulate innate immune pathways necessary for robust downstream immune responses [53]. Immune simulation results provided additional support for the immunogenic potential of the construct, predicting coordinated activation of humoral and cellular immune compartments. The simulated immune response showed early antigen clearance, class switching from IgM to IgG antibodies, expansion of memory B and T cell populations, and elevated cytokine production, including IFN-γ and IL-2. Such immune signatures are consistent with the development of long-term protective immunity and further validate the rational design of the vaccine construct [54].

Importantly, the in vivo validation in albino mice corroborated the in silico predictions. Mice immunized with the alum-adjuvanted MEV-DV vaccine exhibited detectable antibody responses as early as day 7, with peak hemagglutination inhibition (HI) titers observed at day 21 following booster immunization. The magnitude and kinetics of the antibody response were statistically comparable to those elicited by a commercial inactivated dengue vaccine, demonstrating immunological equivalence. The PBS control group remained seronegative, confirming the antigen specificity of the response. Moreover, no adverse reactions or behavioral abnormalities were observed in vaccinated animals, indicating that the MEV-DV construct is well tolerated and safe in the tested model [55]. These findings are particularly significant in the context of ongoing challenges associated with tetravalent dengue vaccines. Current licensed vaccines have shown variable efficacy across serotypes and age groups and have raised safety concerns related to ADE in seronegative individuals [56, 57]. The epitope-based strategy employed in this study offers a promising alternative by focusing on conserved, non-pathogenic regions of the virus and by excluding whole viral proteins that may contribute to imbalanced immune responses. Such precision vaccine design may reduce the likelihood of inducing non-neutralizing antibodies and thereby lower the risk of ADE.

Nevertheless, this study has several limitations that warrant consideration. While the HI assay demonstrated strong humoral responses, neutralization assays against live virus were not performed, and therefore the protective efficacy of the induced antibodies cannot be fully established. Additionally, cellular immune responses were inferred from immune simulation and epitope prediction but were not directly measured experimentally through assays such as ELISpot or flow cytometry. The use of a single mouse model also limits the generalizability of the findings to human immune responses, particularly with respect to HLA diversity and ADE risk. Furthermore, serotype-specific protection and cross-reactivity were not evaluated individually, which will be important for future development [58].

Future studies should focus on validating the neutralizing capacity of the induced antibodies, assessing cellular immune responses in greater detail, and conducting viral challenge experiments to confirm protective efficacy. Evaluation in additional animal models, including humanized mice or non-human primates, will be critical for assessing translational relevance and safety. Moreover, exploring alternative delivery platforms such as mRNA, viral vectors, or nanoparticle-based formulations could further enhance immunogenicity and stability. Ultimately, integrating this capsid-based MEV-DV construct into a broader multivalent or booster vaccination strategy may provide a next-generation solution for dengue prevention [59].

This study demonstrates that a conserved capsid-based multi-epitope vaccine designed through reverse vaccinology and immunoinformatics can elicit strong and sustained immune responses and is comparable in performance to a commercial dengue vaccine in a murine model. By combining computational design with experimental validation, this work highlights the power of rational vaccine development approaches and provides a strong foundation for further preclinical advancement of MEV-DV as a promising dengue vaccine candidate.

### Limitations and future directions

Although the present study provides strong computational and experimental support for the MEV-DV multi-epitope vaccine, several limitations should be acknowledged. First, while the mouse immunogenicity data demonstrated robust antibody production, the study did not include viral neutralization assays or challenge experiments, which are essential to confirm the protective efficacy of the induced antibodies against live DENV. Second, the evaluation focused solely on humoral immunity; detailed profiling of cellular immune responses, including T-cell activation, cytokine secretion, and memory cell persistence, remains necessary to fully understand the vaccine’s immunological potential. Third, the study utilized a single animal model, which may not fully replicate human immune responses, and serotype-specific responses were not assessed in the context of potential cross-reactivity or ADE. Additionally, large-scale production feasibility, long-term stability, and adjuvant optimization were not explored.

Crucially, the experimental evaluation in an albino mouse model validated the computational predictions. The MEV-DV vaccine induced detectable antibody responses as early as day 7, rising sharply by day 14 and peaking on day 21. This timeline closely reflects the in silico immune simulation results, which predicted early antigen clearance and rapid induction of IgG responses. The humoral response produced by MEV-DV was statistically comparable to that induced by a commercial inactivated dengue vaccine across all evaluated time points (*p* > 0.05). Notably, MEV-DV produced slightly earlier antibody responses and maintained stable HI titers through day 28, whereas the reference vaccine exhibited a slightly broader response range. These comparable results confirm that the computationally derived construct can function similarly to an established commercial benchmark. The PBS group remained seronegative throughout the study, confirming that antibody production in vaccinated groups was antigen-specific. Furthermore, no adverse reactions or abnormalities were observed in any of the immunized mice, supporting the safety and tolerability of the MEV-DV formulation.

These findings highlight that the combination of conserved capsid epitopes, structurally stable design, strong innate receptor binding, robust predicted immune activation, and experimentally validated antibody responses positions MEV-DV as a strong contender for a serotype-specific dengue vaccine. Unlike tetravalent formulations that risk imbalanced immune responses leading to ADE, a serotype-specific approach such as this has the potential to provide safer targeted protection particularly in regions where DENV is predominant or in individuals at risk of secondary infection. The study demonstrates the practical value of integrating reverse vaccinology, immunoinformatics, structural biology, and in vivo validation to accelerate vaccine development. MEV-DV’s performance in mice warrants further evaluation through neutralization assays, cellular immunity profiling, and viral challenge experiments to determine its protective efficacy against DENV infection.

Future studies should aim to validate the protective efficacy of the MEV-DV vaccine through comprehensive in vitro neutralization assays and in vivo viral challenge experiments. To further characterize its immunological profile, detailed analyses of T-cell responses, cytokine signaling, and long-term memory development are needed. Evaluating the vaccine in additional animal models, including humanized mice or non-human primates, will help assess translational relevance and potential ADE-related safety concerns. Moreover, exploring alternative delivery platforms such as mRNA, viral vectors, or nanoparticle-based formulations may enhance immunogenicity and stability. Ultimately, integrating MEV-DV into a multivalent platform could support the development of a next-generation, serotype-tailored dengue vaccine.

## Conclusion

This study successfully integrates reverse vaccinology, immunoinformatics, structural modeling, and in vivo experimentation to develop a promising multi-epitope vaccine candidate targeting Dengue Virus Serotype 1–4. The designed MEV-DV construct demonstrated strong antigenicity, structural stability, favorable physicochemical properties, and potent predicted interactions with innate immune receptors, all of which were supported by robust immune simulation outputs. Importantly, experimental validation in a mouse model confirmed early, sustained, and statistically comparable antibody responses to a commercial dengue vaccine, with no adverse effects observed. By focusing on conserved capsid epitopes, MEV-DV offers a targeted, potentially safer alternative to tetravalent dengue vaccines and represents a strong foundation for further preclinical evaluation. Future studies involving neutralization assays, cellular immunity profiling, and challenge experiments will be essential to advance this candidate toward translational development.

## Supplementary Information

Supplementary Material 1.

## Data Availability

Overall data of this research paper is present in the manuscript and supplementary file.
