# Supplementary material for: A conserved capsid-based multi-epitope vaccine targeting dengue virus serotypes (DENV1–4): an integrated computational and in vivo study
Source: BMC Immunol. 2026 May 27;27:68. doi: 10.1186/s12865-026-00857-1 (PMC13404919; doi:10.1186/s12865-026-00857-1)
Supplement: Supplementary file 1 — Supplementary Material 1. [file 12865_2026_857_MOESM1_ESM.docx]

**Table S1:** The top 6 MHCI epitopes with immunogenic properties and respective alleles.

| **Protein IDs** | **Epitopes** | **Antigenicity** | **Allergenicity** | **Alleles** | **Median binding percentile** | **Net MHC panel scores** | **Toxicity** |
| --- | --- | --- | --- | --- | --- | --- | --- |
| NP_059433.1 | KTQAGVVRR | 0.9523 | Non | HLA-A*31:01 | 0.01 | 0.9609 | Non-Toxin |
| NP_739591.2 | RGLLLDTMW | 0.8632 | Non | HLA-B*58:01 | 3.2 | 0.8148 | Non-Toxin |
| A0A217EIR2 | ATGPITTLW | 1.1256 | Non | HLA-B*58:01 | 0.01 | 0.9977 | Non-Toxin |
| ARO34439.1 | TLLCLIPTV | 1.1774 | Non | HLA-A*02:01 | 0.1 | 0.7644 | Non-Toxin |
| ARO34441.1 | CLIPTVMAF | 0.9626 | Non | HLA-A*02:01 | 2.2 | 0.0470 | Non-Toxin |
| ARO34442.1 | STITLLCLI | 1.2445 | Non | HLA-A*02:01 | 3.9 | 0.0167 | Non-Toxin |

**Table S2:** The top 6 MHCII epitopes with immunogenic properties and respective alleles.

| **Protein IDs** | **Epitopes** | **Antigenicity** | **Allergenicity** | **Alleles** | **Median binding percentile** | **Net MHC panel scores** | **Toxicity** |
| --- | --- | --- | --- | --- | --- | --- | --- |
| NP_059433.1 | VSGALLGK  AVHSGAQ | 0.9021 | Non | HLA-DRB1*08:02 | 52 | 0.0165 | Non-Toxin |
| NP_739591.2 | RNYNQMR  VGRRGTRH | 1.1022 | Non | HLA-DRB5*01:01 | 95 | 0.0952 | Non-Toxin |
| A0A217EIR2 | EHNSTWH  YDDENPYK | 0.8562 | Non | HLA-DRB3*01:01 | 64 | 0.1075 | Non-Toxin |
| ARO34439.1 | LAFITFLR  VLSIPPT | 0.8721 | Non | HLA-DRB1*01:01 | 14 | 0.1028 | Non-Toxin |
| ARO34441.1 | ITLLCLIP  TVMAFSL | 1.2470 | Non | HLA-DRB1*01:01 | 44 | 0.0090 | Non-Toxin |
| ARO34442.1 | NILNGRK  RSTITLLC | 1.1476 | Non | HLA-DRB1*01:01 | 97 | 0.0003 | Non-Toxin |

**Table S3:** The top 6 B-cell epitopes with immunogenic properties.

| **Protein ID** | **Epitope**  **Sequence** | **Antigenicity** | **Hydrophobicity** | **IL-4 Score** | **IL-10 Score** | **IFN-γ score** | **Toxicity** |
| --- | --- | --- | --- | --- | --- | --- | --- |
| NP_059433.1 | PLMIVAK  ERGRPLLT | 0.8325 | 62.67% | 0.11 | 0.05 | 0.41 | Non-Toxin |
| NP_739591.2 | IPTVMAFS  LSTRDGEP | 1.1494 | 60% | 0.09 | 0.12 | 0.18 | Non-Toxin |
| A0A217EIR2 | PLMIVAK  HERGRPLLM | 0.8214 | 42.5% | 0.07 | 0.06 | 0.47 | Non-Toxin |
| ARO34439.1 | KCTLIAMD  LGEMCDDA | 0.9335 | 39.5% | 0.12 | 0.09 | 0.21 | Non-Toxin |
| ARO34441.1 | TTEGINKC  TLIAMDLE | 0.7254 | 42% | 0.08 | 0.05 | 0.15 | Non-Toxin |
| ARO34442.1 | FSLSTRDG  EPLMIVAS | 1.3251 | 60.5% | 0.06 | 0.03 | 0.08 | Non-Toxin |

**Table S4:** Conformation of Discontinuous B-cell Epitopes in Refined Vaccine

| **No** | **Residues** | **Number of residues** | **ElliPro Score** |
| --- | --- | --- | --- |
| 1. | A:A62, A:T64, A:L65, A:L66, A:C67, A:L68, A:I69, A:P70, A:T71, A:V72, A:A73, A:A74, A:Y75, A:L76, A:L77, A:F78, A:K79, A:T80, A:T81, A:E82, A:G83, A:I84, A:A85, A:A86, A:Y87, A:I88, A:T89, A:F90, A:L91, A:R92, A:V93, A:L94 | 32 | 0.811 |
| 2. | A:M120, A:A121, A:A122, A:Y123, A:L124, A:A125, A:F126, A:I127, A:T128, A:F129, A:L130, A:R131, A:V132, A:G133, A:P134, A:G135, A:P136, A:G137, A:K138, A:K139, A:N140, A:K141, A:A142, A:I143, A:K144, A:I145, A:L146, A:I147, A:G148, A:F149, A:R150, A:K151, A:E152, A:G153, A:P154, A:G155, A:P156, A:G157 | 38 | 0.794 |
| 3. | A:K253, A:K254, A:P255, A:L256, A:M257, A:I258, A:V259, A:A260, A:K261, A:E262, A:R263, A:G264, A:R265, A:P266, A:L267, A:L268, A:F269, A:K270, A:K271, A:I272, A:P273, A:T274, A:V275, A:M276, A:A277, A:M292, A:I293, A:V294, A:A295, A:K296, A:H297, A:E298, A:R299, A:G300, A:R301, A:P302, A:L303, A:L304, A:K308, A:C309, A:T310, A:L311, A:I312, A:A313, A:M314, A:D315, A:L316, A:G317, A:E318, A:M319, A:C320, A:D321, A:D322, A:K324, A:K325, A:T326, A:T327, A:E328, A:G329, A:I330, A:K332 | 61 | 0.713 |
| 4. | A:G1, A:I2, A:I3, A:N4, A:T5, A:Q7, A:K8, A:G16, A:R17, A:C18, A:A19, A:V20, A:L21, A:S22, A:C23, A:L24, A:P25, A:K26, A:E27, A:Q29, A:I30, A:G31, A:K32, A:C33, A:S34, A:T35, A:R36, A:G37, A:R38, A:K39, A:C40, A:C41, A:R42 | 33 | 0.7 |
| 5. | A:A362, A:A363, A:K364 | 3 | 0.655 |
| 6. | A:L212, A:G213, A:P214, A:G215, A:P216, A:G217, A:K219, A:R220 | 8 | 0.611 |
| 7. | A:S347, A:R349, A:D350, A:G351, A:E352, A:M355, A:I356, A:K359 | 8 | 0.547 |

**Table S5:** The full Alleles list used for epitope prediction

| MHCI Alleles | MHCII Alleles |
| --- | --- |
| HLA-A*01:01, 9  HLA-A*02:01, 9  HLA-A*02:06, 9  HLA-A*03:01, 9  HLA-A*11:01, 9  HLA-A*23:01, 9  HLA-A*24:02, 9  HLA-A*25:01, 9  HLA-A*26:01, 9  HLA-A*29:02, 9  HLA-A*30:01, 9  HLA-A*30:02, 9  HLA-A*31:01, 9  HLA-A*32:01, 9  HLA-A*68:01, 9  HLA-A*68:02, 9  HLA-B*07:02, 9  HLA-B*08:01, 9  HLA-B*14:02, 9  HLA-B*15:01, 9  HLA-B*15:02, 9  HLA-B*18:01, 9  HLA-B*27:05, 9  HLA-B*35:01, 9  HLA-B*35:03, 9  HLA-B*35:03, 9  HLA-B*38:01, 9  HLA-B*39:01, 9  HLA-B*40:01, 9  HLA-B*40:02, 9  HLA-B*44:03, 9  HLA-B*46:01, 9  HLA-B*48:01, 9  HLA-B*51:01, 9  HLA-B*53:01, 9  HLA-B*57:01, 9  HLA-B*58:01, 9  HLA-B*58:02, 9  HLA-C*03:03, 9  HLA-C*04:01, 9  HLA-C*05:01, 9  HLA-C*06:02, 9  HLA-C*07:01, 9  HLA-C*07:02, 9  HLA-C*08:02, 9  HLA-C*12:03, 9  HLA-C*14:02, 9  HLA-C*14:02, 9  HLA-C*15:02, 9  HLA-E*01:01, 9  HLA-E*01:03, 9 | DRB1*01:01  DRB1*03:01  DRB1*04:01  DRB1*04:04  DRB1*04:05  DRB1*07:01  DRB1*08:02  DRB1*09:01  DRB1*11:01  DRB1*12:01  DRB1*13:02  DRB1*15:01  DRB3*01:01  DRB4*01:01  DRB5*01:01  DRB1*01:01  DRB1*03:01  DRB1*04:01  DRB1*04:04  DRB1*04:05  DRB1*07:01  DRB1*08:02  DRB1*09:01  DRB1*11:01  DRB1*12:01  DRB1*13:02  DRB1*15:01  DRB3*01:01  DRB4*01:01  DRB5*01:01  DRB1*01:01  DRB1*03:01  DRB1*04:01  DRB1*04:04  DRB1*04:05  DRB1*07:01  DRB1*08:02  DRB1*09:01  DRB1*11:01  DRB1*12:01  DRB1*13:02  DRB1*15:01  DRB3*01:01  DRB4*01:01  DRB5*01:01 |


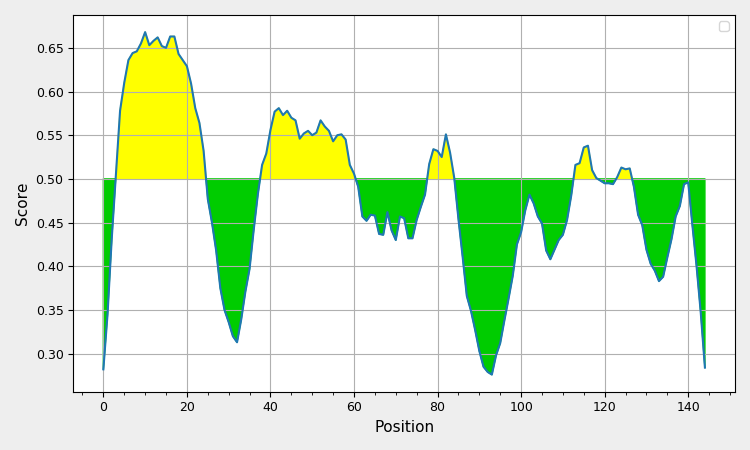


**Figure S1:** The figure shows the binding score profile of a Capsid Protein (ARO34439.1) along its position, with yellow areas indicating scores above 0.5 and green areas indicating scores below 0.5


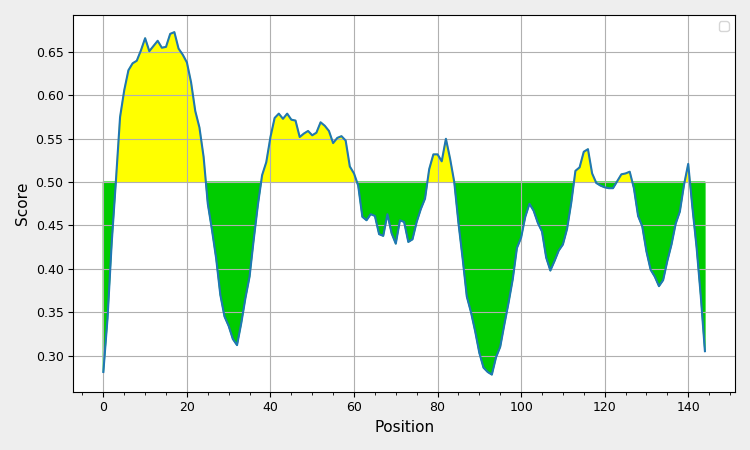


**Figure S2:** The figure shows the binding score profile of a Capsid Protein (ARO34441.1) along its position, with yellow areas indicating scores above 0.5 and green areas indicating scores below 0.5


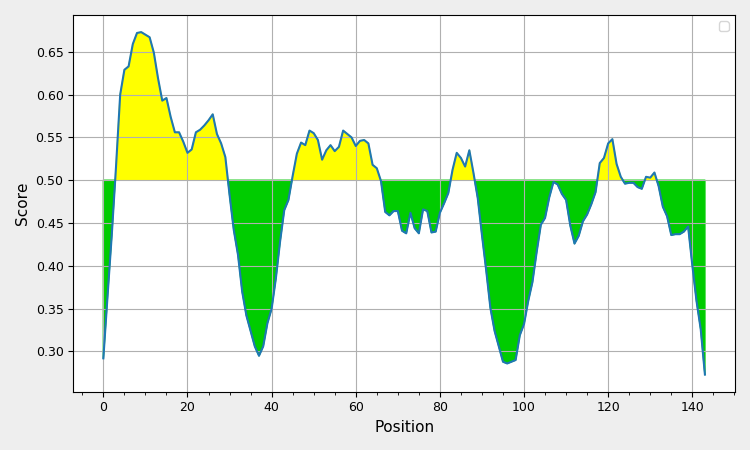


**Figure S3:** The figure shows the binding score profile of a Capsid Protein (ARO34442.1) along its position, with yellow areas indicating scores above 0.5 and green areas indicating scores below 0.5


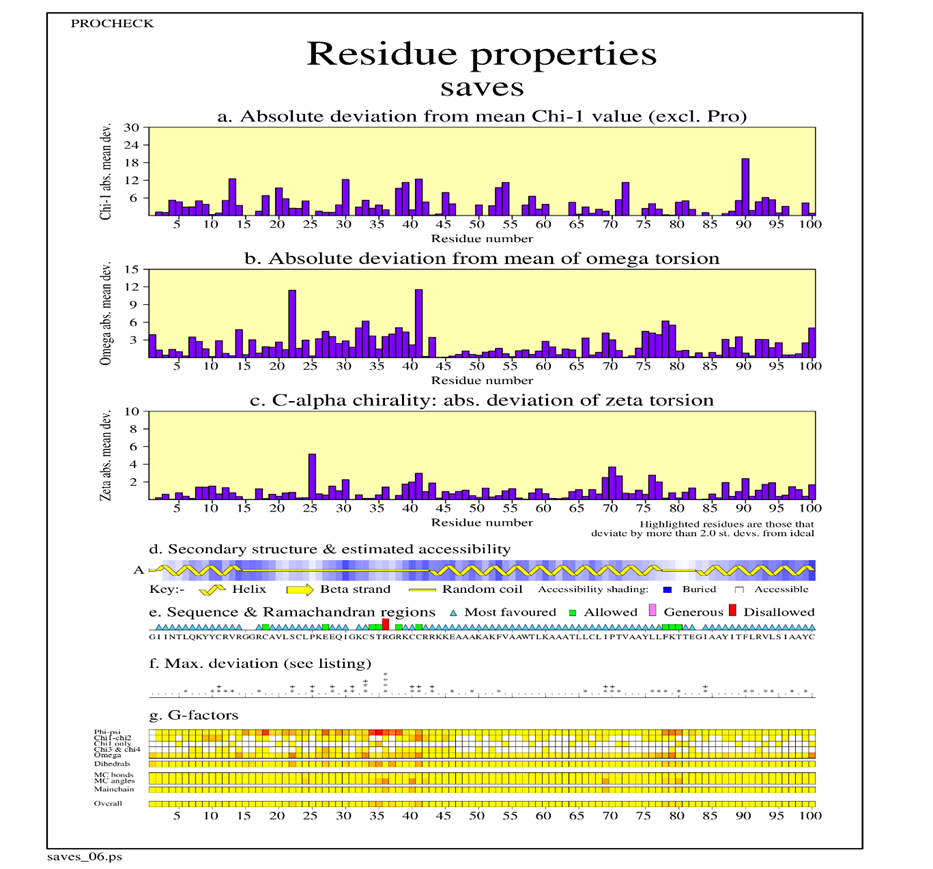


**Figure S4:** Vaccine construct residue properties with absolute deviation from mean chi_1 value, omega torsion, zeta torsion, Ramachandran regions and G-factors.
